# Discovery and functional characterisation of a luqin-type neuropeptide signalling system in a deuterostome

**DOI:** 10.1038/s41598-018-25606-2

**Published:** 2018-05-08

**Authors:** Luis Alfonso Yañez-Guerra, Jérôme Delroisse, Antón Barreiro-Iglesias, Susan E. Slade, James H. Scrivens, Maurice R. Elphick

**Affiliations:** 1 Queen Mary University of London, School of Biological & Chemical Sciences, Mile End Road, London, E1 4NS UK; 2 Waters/Warwick Centre for BioMedical Mass Spectrometry and Proteomics, School of Life Sciences, University of Warwick, Coventry, CV4 7AL UK; 3 Present Address: University of Mons, Research Institute for Biosciences, Biology of Marine Organisms and Biomimetics, 23 Place du Parc, Mons, 7000 Belgium; 4 Present Address: Department of Functional Biology, CIBUS, Faculty of Biology, Universidade de Santiago de Compostela, 15782 Santiago de Compostela, Spain; 5 Present Address: Waters Corporation, Stamford Avenue, Altrincham Road, Wilmslow, SK9 4AX UK; 6 Present Address: School of Science, Engineering & Design, Stephenson Street, Teesside University, Middlesbrough TS1 3BX, Tees Valley TS1 3BA UK

## Abstract

Neuropeptides are diverse and evolutionarily ancient regulators of physiological/behavioural processes in animals. Here we have investigated the evolution and comparative physiology of luqin-type neuropeptide signalling, which has been characterised previously in protostomian invertebrates. Phylogenetic analysis indicates that luqin-type receptors and tachykinin-type receptors are paralogous and probably originated in a common ancestor of the Bilateria. In the deuterostomian lineage, luqin-type signalling has been lost in chordates but interestingly it has been retained in ambulacrarians. Therefore, here we characterised luqin-type signalling for the first time in an ambulacrarian – the starfish *Asterias rubens* (phylum Echinodermata). A luqin-like neuropeptide with a C-terminal RWamide motif (ArLQ; EEKTRFPKFMRW-NH_2_) was identified as the ligand for two luqin-type receptors in *A*. *rubens*, ArLQR1 and ArLQR2. Furthermore, analysis of the expression of the ArLQ precursor using mRNA *in situ* hybridisation revealed expression in the nervous system, digestive system and locomotory organs (tube feet) and *in vitro* pharmacology revealed that ArLQ causes dose-dependent relaxation of tube feet. Accordingly, previous studies have revealed that luqin-type signalling regulates feeding and locomotor activity in protostomes. In conclusion, our phylogenetic analysis combined with characterisation of luqin-type signalling in a deuterostome has provided new insights into neuropeptide evolution and function in the animal kingdom.

## Introduction

Neuropeptides play a central role in the control of diverse physiological processes and behaviours in animals. Furthermore, neuropeptides are evolutionarily ancient mediators of neuronal signalling and a large number of different neuropeptide signalling pathways were already present in the common ancestor of protostomes and deuterostomes^1–3^. The discovery of neuropeptide signalling systems has been enabled by a variety of experimental strategies^4^. The luqin-type neuropeptide system that is the focus of this study was first discovered using a molecular biological approach where the objective was to identify neuropeptides expressed in the L5 neuron of the abdominal ganglion in the mollusc *Aplysia californica* that are immunoreactive with antibodies to the neuropeptide FMRFamide. A cDNA encoding a novel precursor protein comprising a peptide with the predicted C-terminal tetrapeptide sequence QGRFamide was discovered^5^. Subsequently, the mature peptide derived from this precursor was identified biochemically as APSWRPQGRFamide and named luqin (LQ) because it is expressed in the Left Upper Quadrant cells of the abdominal ganglion in *A*. *californica*^6^. However, prior to this a homolog of luqin (SGQWRPQGRFamide) was discovered in the snail *Achatina fulica* and named Achatina Cardio-Excitatory Peptide (ACEP-1) on account of its effect in potentiating the beat of the heart ventricle in this species. Furthermore, ACEP-1 was also found to have excitatory effects on neurons and muscles involved in feeding behaviour in *A*. *fulica*^7^. A third luqin-type peptide (TPHWRPQGRFamide) was discovered in the pond snail *Lymnaea stagnalis* and named *Lymnaea* cardioexcitatory-peptide (LyCEP) because it increases the beating frequency of auricle preparations from this species^8^. Concomitant with the discovery of LyCEP, an orphan G-protein coupled receptor (GRL106) was identified as the receptor for this peptide and thus the first receptor for luqin-type peptides was discovered^8^. More recently, analysis of genomic sequence data enabled the discovery of luqin-type neuropeptides in the annelid *Capitella teleta*^9^. Furthermore, the receptor for the luqin-type peptide WRPGRFamide has been identified in the annelid *Platynereis dumerilii*^10,11^.

Phylogenetic analysis of G-protein coupled neuropeptide receptors has revealed that molluscan luqin receptors are orthologs of receptors for arthropod neuropeptides that have a C-terminal RYamide motif – ‘RYamides’^3^. Additional evidence that lophotrochozoan luqins and arthropodan RYamides are orthologous was provided by similarity-based clustering methods^1^ and the identification of a conserved motif comprising two cysteine residues in the C-terminal region of luqin/RYamide-type precursor proteins^3^. Thus, luqin-type neuropeptides from lophotrochozoan protostomes (molluscs, annelids) and RYamides from ecdysozoan protostomes (arthropods, nematodes) were unified as members of a bilaterian family of luqin/RYamide-type neuropeptides^1,3^.

RYamides were first identified in crustaceans^12–14^ and subsequently RYamide precursor proteins and RYamide receptors were discovered in insects^15–17^. Analysis of RYamide expression in *Drosophila melanogaster* revealed that it is expressed by neurons that innervate the rectal papillae, organs that mediate water re-absorption in flies. Consistent with this expression pattern, injection of female mosquitoes with RYamides delays postprandial diuresis^18^. RYamides also suppress feeding motivation and sucrose consumption in the blow fly *Phormia regina*^19^ and evidence of a role in regulation of feeding has also been obtained in the crustacean *Marsupenaeus japonicus*, where expression of the RYamide precursor gene is downregulated during starvation^20^. Recently, a detailed molecular and functional characterisation of luqin/RYamide-type neuropeptide signalling in the nematode *Caenorhabditis elegans* has been reported. Consistent with the findings from arthropods, luqin/RYamide-type signalling suppresses feeding behaviour in *C*. *elegans* whilst also influencing egg-laying, lifespan and locomotor activity^21^.

Analysis of the phylogenetic distribution of luqin/RYamide-type receptors has revealed the presence of orthologs in ambulacrarians (hemichordates and echinoderms) but not in vertebrates and other chordates (urochordates and cephalochordates)^1,3^. Thus, the evolutionary origin of luqin/RYamide-type neuropeptide signalling can be traced to common ancestor of protostomes and deuterostomes, but with subsequent loss in the chordate lineage. Furthermore, consistent with this conclusion, precursor proteins comprising candidate ligands for luqin/RYamide-type receptors have been identified in ambulacrarians but not in chordates^1,22^.

Luqin/RYamide-type precursors in ambulacrarians comprise a neuropeptide with a putative C-terminal RWamide motif, which contrasts with the RFamide/RYamide motif found in protostomian luqin/RYamide-type neuropeptides. However, there have been no experimental studies on luqin/RWamide-type neuropeptide signalling in deuterostomes. The objective of this study was to begin to fill this gap in our knowledge and to accomplish this we selected the starfish *Asterias rubens* (phylum Echinodermata) as an experimental system, building upon a growing body of data on neuropeptide signalling that have been obtained from this species^22,23^. Because of its phylogenetic position as a non-chordate deuterostome, *A*. *rubens* and other echinoderms can provide key insights into the evolution of neuropeptide signalling systems. This was illustrated recently with deorphanisation of echinoderm neuropeptide receptors facilitating reconstruction of the evolutionary history of neuropeptide-S/crustacean cardioactive peptide (CCAP)-type signalling^24^ and gonadotropin-releasing hormone (GnRH)/corazonin-type signalling^25^. Furthermore, the pentaradial symmetry of adult echinoderms provides a unique context for comparative analysis of neuropeptide function in the animal kingdom^26–28^.

Here we report the first biochemical, anatomical and pharmacological characterisation of luqin/RWamide-type neuropeptide signalling in a deuterostome, the starfish *A*. *rubens*, providing new insights into the evolution and comparative physiology of neuropeptide signalling systems in the animal kingdom.

## Results

### Sequencing of a luqin-type precursor and a luqin-type neuropeptide in *A*. *rubens*

**Figure 1:**
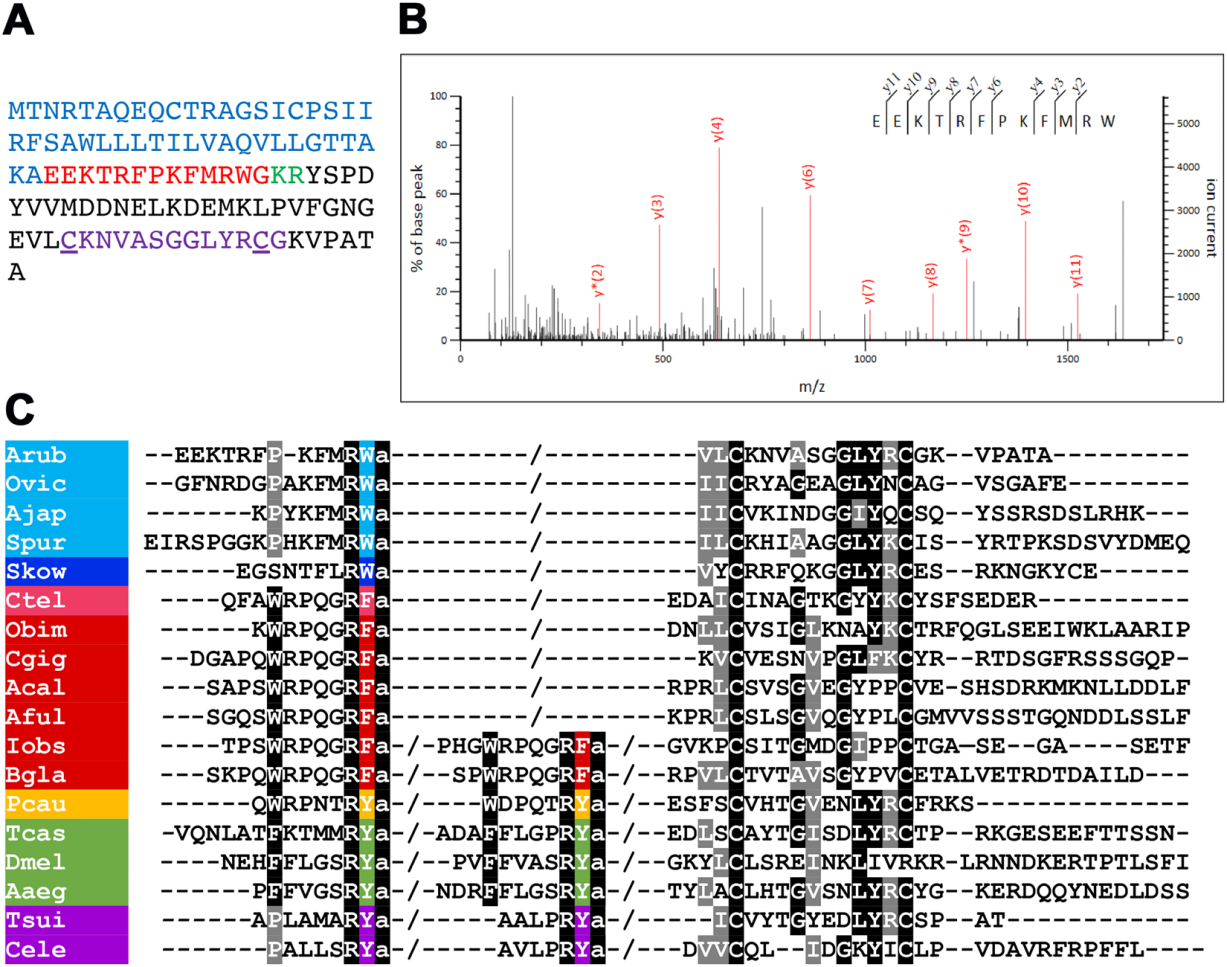
*Asterias rubens* luqin-type precursor (ArLQP) and luqin-type neuropeptide (ArLQ). (**A**) Amino acid sequence of ArLQP, with the predicted signal peptide shown in blue, the predicted luqin-type neuropeptide shown in red and a potential dibasic cleavage site shown in green. Shown in purple is a region of the precursor near to the C-terminus containing two cysteine residues (underlined), which is a conserved feature of luqin-type precursors (see alignment in (**C**). (**B**) Mass spectrometric identification of ArLQ (EEKTRFPKFMRW-NH_2_) from an acetic acid extract of radial nerve cords from *A*. *rubens*. Annotated MS/MS spectrum indicated in red, with ions matched to the sequence used for Mascot scoring. A triply charged peptide was identified with a Mascot ions score of 45, expect value of 0.00018 based on UniProt/TrEMBL protein database filtered for taxon identifier 7586 (Echinodermata, 70,885 sequences) with a precursor mass error of 2.7 ppm. (**C**) Alignment of the N-terminal neuropeptide-containing region and C-terminal region of ArLQP with corresponding regions of other luqin/RYamide-type precursor proteins. Conserved residues are highlighted in black or grey. The C-terminal residues of the luqin/RYamide-type peptides and species names are highlighted in phylum-specific colours: light blue (Echinodermata), dark blue (Hemichordata), pink (Annelida), red (Mollusca), yellow (Priapulida), green (Arthropoda) and purple (Nematoda). Species names are as follows: Arub (*Asterias rubens*), Ovic (*Ophionotus victoriae*), Ajap (*Apostichopus japonicus*), Spur (*Strongylocentrotus purpuratus*), Skow (*Saccoglossus kowalevskii*), Ctel (*Capitella teleta*), Obim (*Octopus bimaculoides*), Cgig (*Crassostrea gigas*), Acal (*Aplysia californica*), Aful (*Achatina fulica*), Iobs (*Ilyanasa obsoleta*), Bgla (*Biomphalaria glabrata*), Pcau (*Priapulus caudatus*), Tcas (*Tribolium castaneum*), Dmel (*Drosophila melanogaster*), Aaeg (*Aedes aegypti*), Tsui (*Trichuris suis*), Cele (*Caenorhabditis elegans*). The accession numbers of the sequences included in this alignment are listed in supplementary Table 2.

Cloning and sequencing of a cDNA encoding a luqin-type precursor protein (ArLQP) confirmed a previously reported sequence assembled from *A*. *rubens* transcriptome data^22^. ArLQP is a 106-residue protein, including a predicted 44-residue N-terminal signal peptide and a predicted luqin-like peptide sequence (EEKTRFPKFMRWG), followed by a dibasic cleavage site (KR) (Fig. 1A). Analysis of *A*. *rubens* radial nerve cord extracts using mass spectrometry confirmed the presence of the predicted luqin-like peptide, with post-translational conversion of the C-terminal glycine to an amide group (ArLQ; EEKTRFPKFMRW-NH_2_; Fig. 1B). Alignment of ArLQP with luqin/RYamide-type precursors from other species revealed several similarities. ArLQP comprises a single luqin/RYamide-like neuropeptide, a feature shared with luqin-type precursors in other echinoderms and in hemichordates and annelids (Fig. 1C). This contrasts with precursor proteins in ecdysozoans (insects, nematodes and the priapulid *Priapulus caudatus*), which comprise two luqin-like RYamides (Fig. 1C). Interestingly, precursors comprising either one or two luqin-type peptides are found in molluscs (Fig. 1C). A distinctive feature of luqin/RYamide-type precursors that has been reported previously^1,3^ are a pair of cysteine residues located in the C-terminal region and separated by ten other amino acid residues. As can be seen in the alignment in Fig. 1C, this feature is conserved in ArLQP and in the majority of luqin/RYamide precursors from other species, with the exception of *D*. *melanogaster* where one of the cysteines is replaced with an arginine residue and *C*. *elegans* where the two cysteines are separated by eight amino acid residues. Phylogenetic analysis of luqin/RYamide-type precursors revealed that they cluster in three distinct clades: deuterostomian precursors comprising a neuropeptide with a C-terminal RWamide motif, lophotrochozoan precursors comprising one or two neuropeptides with a C-terminal RFamide motif and ecdysozoan precursors comprising two neuropeptides with a C-terminal RYamide motif (Fig. 1C; Supplementary Fig. 1).

### Identification of two G-protein coupled receptors in *A*. *rubens* that are activated by ArLQ

**Figure 2:**
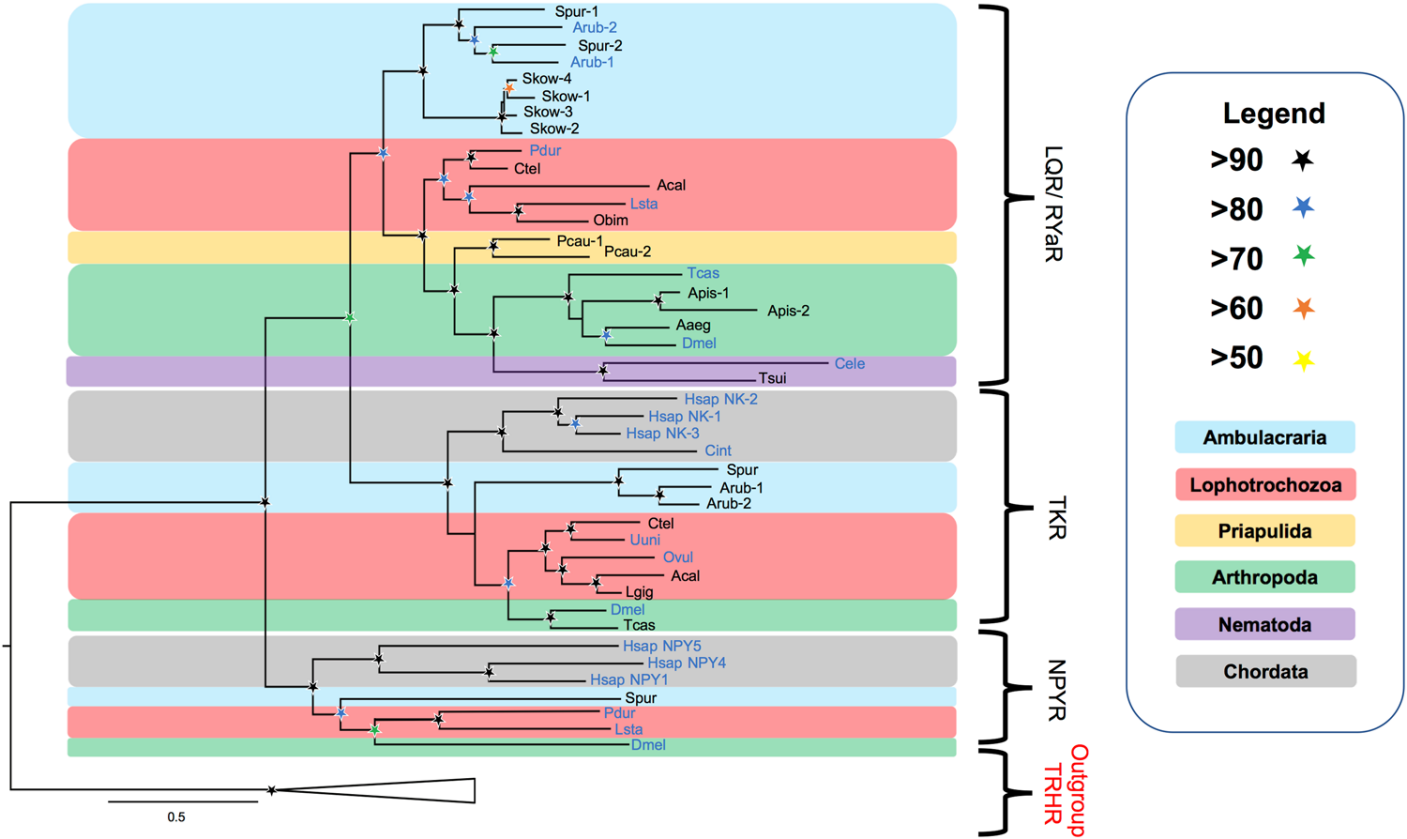
Phylogenetic tree showing luqin/RYamide-type receptors from bilaterians, including the starfish *A*. *rubens*, and other closely related neuropeptide receptors. The tree, which was generated in PHYML 3.0 ^42^ using the Maximum likelihood method^47,48^, comprises three distinct receptor clades – luqin/RYamide-type receptors, tachykinin-type receptors, neuropeptide-Y-type receptors, with TRH-type receptors as an outgroup. Taxa are colour-coded and bootstrap support (1000 replicates^49^) for clades is represented with coloured stars, as explained in the key. Species in which the peptide ligands that activate luqin/RYamide-type receptors have been identified experimentally are shown with blue lettering. Species names are as follows: Aaeg (*Aedes aegypti*), Acal *(Aplysia californica)*, Apis *(Acyrthosiphon pisum)*, Arub *(Asterias rubens)*, Cele *(Caenorhabditis elegans)*, Cint (*Ciona intestinalis*), Ctel *(Capitella teleta)*, Dmel *(Drosophila melanogaster)*, Dpul (*Daphnia pulex)*, Hsap (*Homo sapiens*), Lgig *(Lottia gigantea)*, Lsta (*Lymnaea stagnalis*), Obim (*Octopus bimaculoides*), Ovul (*Octopus vulgaris*), Pcau (*Priapulus caudatus*), Pdum (*Platynereis dumerilii*), Skow (*Saccoglossus kowalevskii*), Spur (*Strongylocentrotus purpuratus*), Tcas *(Tribolium castaneum)*, Tsui (*Trichuris suis*), Uuni (*Urechis unicinctus*). The accession numbers of the sequences included in this phylogenetic tree are listed in the supplementary Table 3.

To identify candidate receptors for ArLQ, we performed BLAST analysis of *A*. *rubens* neural transcriptome sequence data using the deorphanised *P*. *dumerilii* Luqin receptor as a query^10^. Two contigs (1121303 and 1122311) encoding receptors comprising 347 and 388 amino acid residues were identified and named ArLQR1 and ArLQR2, respectively. Analysis of the sequences of ArLQR1 and ArLQR2 using Protter^29^ revealed the presence of seven transmembrane domains, a feature common to G-protein coupled receptors^30^ (Supplementary Figs 2 and 3). Phylogenetic analysis of relationships of ArLQR1 and ArLQR2 with luqin/RYamide-type receptors and with other receptors that are closely related to luqin/RYamide-type receptors, including tachykinin (TK)-type receptors and neuropeptide Y/F (NPY/F) receptors, demonstrated that ArLQR1 and ArLQR2 are orthologs of the luqin/RYamide-type receptors that have been characterised in other phyla. Thus, in a tree rooted with thyrotropin-releasing hormone (TRH)-type receptors as an outgroup there is strong bootstrap support for three distinct clades, a luqin/RYamide receptor clade, a TK receptor clade and a NPY/F receptor clade, and ArLQR1 and ArLQR2 are positioned within the luqin/RYamide receptor clade. Furthermore, ArLQR1 and ArLQR2 are positioned in a branch of the luqin/RYamide receptor clade that includes related receptors from other ambulacrarians – the sea urchin *Strongylocentrotus purpuratus* (phylum Echinodermata) and the acorn worm *Saccoglossus kowalevskii* (phylum Hemichordata) (Fig. 2).

**Figure 3:**
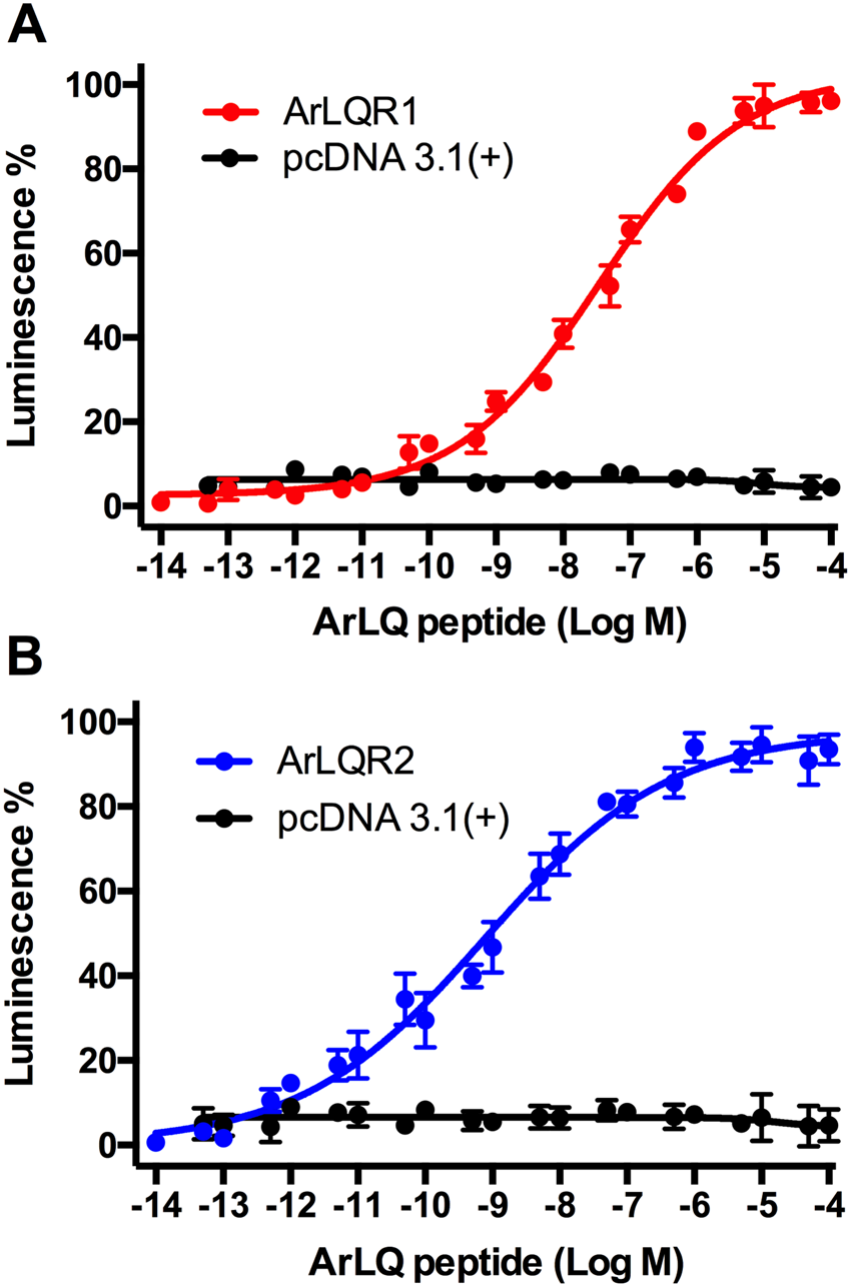
ArLQ acts as a ligand for two *A*. *rubens* G-protein coupled receptors, ArLQR1 and ArLQR2. The graphs show that ArLQ causes dose-dependent activation of ArLQR1 (**A**, red) and ArLQR2 (**B**, blue) expressed in CHO-K1 cells expressing the promiscuous Gα16 protein and a calcium-sensitive bioluminescent GFP-aequorin fusion protein (G5A). Each point represents mean values (± S.E.M.) from at least four independent experiments, with each experiment performed in triplicate. Control experiments where cells were transfected with an empty pcDNA 3.1(+) vector are shown in black. Luminescence is expressed as a percentage of the maximal response observed in each experiment. The EC_50_ values for activation of ArLQR1 and ArLQR2 with ArLQ are 2.4 × 10^−8^ M and 7.8 × 10^−10^ M, respectively.

Having identified ArLQR1 and ArLQR2 as candidate receptors for ArLQ based on phylogenetic analysis, cDNAs encoding these receptors were cloned and then expressed in a CHO-cell line expressing apoaequorin and Gα16 to produce the cell systems CHO-ArLQR1 and CHO-ArLQR2. Synthetic ArLQ (EEKTRFPKFMRW-NH_2_) was then tested as a candidate ligand for these receptors at concentrations ranging from 1 × 10^−4^ M to 1 × 10^−14^ M. ArLQ induced a dose-dependent bioluminescence signal in the CHO-ArLQR1 and CHO-ArLQR2 systems with half-maximal response concentrations (EC_50_) of 2.4 × 10^−8^ M and 7.8 × 10^−10^ M, respectively (Fig. 3). Bioluminescence was observed within 5 s of exposure to ArLQ, after which the signal decreased slowly (Supplementary Fig. 4). Importantly, no response to ArLQ was observed in CHO-cells transfected with the vector alone, demonstrating that the signal observed in CHO-ArLQR1 and CHO-ArLQR2 can be attributed to the transfected receptors (Fig. 3). The specificity of ArLQR1 and ArLQR2 as receptors for ArLQ was further assessed by testing three other neuropeptides from *A*. *rubens* that are evolutionarily related and/or exhibit some C-terminal structural similarity with ArLQ: the Neuropeptide Y-type peptide ArNPY (pQDRSKAMQAERTGQLRRLNPRF-NH_2_)^31^, the tachykinin-like peptide ArTK2 (GGGVPHVFQSGGIF-NH_2_)^22^ and SALMFamide-2 (SGPYSFNSGLTF-NH_2_)^32^. These peptides were tested at concentrations ranging from 1 × 10^−4^ M to 1 × 10^−13^ M but none of them caused activation of the receptors, demonstrating the specificity of ArLQR1 and ArLQR2 as receptors for ArLQ (Supplementary Fig. 5). Thus, we conclude that ArLQ is the ligand for ArLQR1 and ArLQR2 in *A*. *rubens*.

### ArLQP is expressed by cells in the nervous, digestive and locomotor systems in *A*. *rubens*

**Figure 4:**
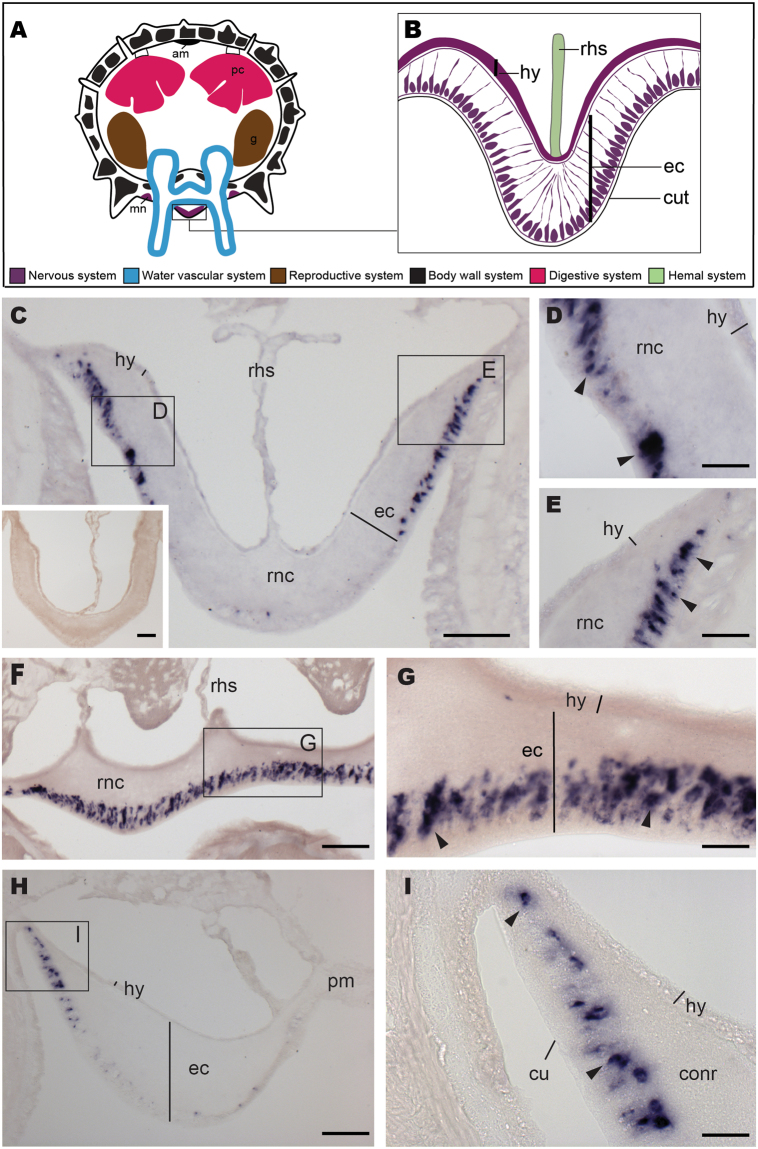
Localisation of ArLQP expression in the nervous system of *A*. *rubens* using mRNA *in situ* hybridisation. (**A**) Schematic showing the anatomy of the starfish arm as seen from a transverse section. (**B**) Schematic showing the anatomy of a radial nerve cord as seen in transverse section. (**C**) Transverse section of a radial nerve cord showing stained cells concentrated in the lateral parts of the ectoneural region. Higher magnification images of the boxed regions are shown in the panels (**D**) and (**E**). The inset shows absence of staining in a transverse section of radial nerve cord incubated with sense probes, demonstrating the specificity of staining observed with antisense probes. (**F**). Longitudinal parasagittal section of a radial nerve cord showing stained cells in the ectoneural region (arrowheads). A higher magnification of the boxed region is shown in the panel (**G**). (**H**) Transverse section of the circumoral nerve ring showing stained cells concentrated the lateral part of the ectoneural region. The boxed region is shown at higher magnification in panel **I**. am, *apical muscle*; conr, *circumoral nerve ring*; cut, *cuticle*; ec, *ectoneural region*; g, *gonads*; hy, *hyponeural region*; mn, *marginal nerve*; pc, *pyloric caeca*; pm, *peristomial membrane*; rhs, *radial hemal sinus*; rnc, *radial nerve cord*. Scale bars: 50 μm in **C**, **C** inset, **F**, **H**; 10 μm in **D**, **E**, **G**, **I**.

**Figure 5:**
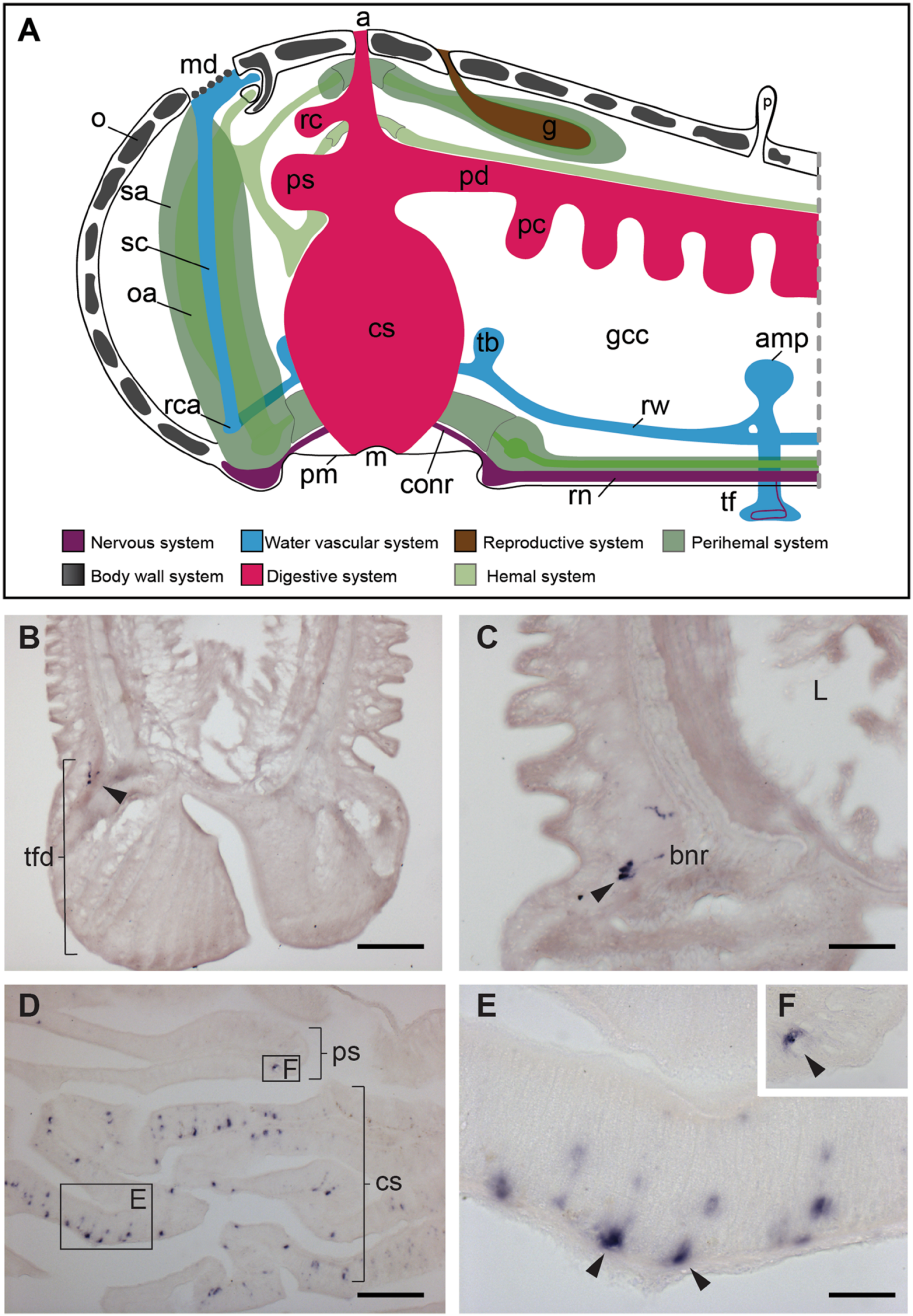
Localisation of ArLQP expression in the tube feet and stomach of *A*. *rubens* using mRNA *in situ* hybridisation. (**A**) Schematic showing the anatomy of the central disk region and an adjoining arm in starfish. (**B**) Longitudinal section of a tube foot showing stained cells (arrowhead) associated with the basal nerve ring in the disk region. (**C**) High magnification image showing stained cells (arrowhead) associated with the basal nerve ring in the disk region of a tube foot. (**D**) Transverse section of the central disk region showing stained cells in the cardiac stomach and pyloric stomach. A higher magnification of the boxed region of the cardiac stomach is shown in (**E**), where stained cells can be seen in the mucosal layer of the cardiac stomach, with some cells (arrowheads) in close proximity to the basi-epithelial nerve plexus. A higher magnification image of a stained cell in the pyloric stomach is shown in (**F**). a, *anus*; amp, *ampullae*; bnr, *basal nerve ring*; conr, *circumoral nerve ring*; cs, *cardiac stomach*; g, *gonad*; gcc, *general coelomic cavity*; l, *lumen*; m, *mouth*; md, *madreporite*; o, *ossicle*; *oa, organ axial;* p, *papillae*; pc, *pyloric caecum*; pd, *pyloric duct*; pm, *peristomial membrane*; ps, *pyloric stomach*; rc, *rectal caecum*; rca, *ring canal*; rn, *radial nerve*; rw, *radial water vascular canal*; *sa, sinus of axial organ;* sc, *stone canal*; tb, *Tiedemann’s bodies*; tf, *tube foot*; tfd, *tube foot disc*. Scale bars: 50 μm in **B**, **D**; 20 μm in **C**; 10 μm in **E** and **F**.

To gain insights into the physiological roles of ArLQ in *A*. *rubens*, the expression pattern of ArLQP was examined using mRNA *in situ* hybridisation. No staining was observed in experiments with sense probes (Fig. 4C inset), demonstrating the specificity of staining observed with antisense probes (Figs 4, 5). Cells expressing ArLQP were detected in the nervous system (Fig. 4) and in the digestive system and tube feet (Fig. 5).

The main components of the nervous system in starfish are the radial nerve cords that are located on the underside of each arm and that are linked by a circumoral nerve ring in the central disk region (Fig. 4A,B; Fig. 5A). Transverse sections of arms revealed stained cells in radial nerve cords, concentrated laterally in the subcuticular epithelium of the ectoneural region (Fig. 4C–E). In longitudinal sections of arms stained cells can be seen along the length of the ectoneural region of the radial nerve cords (Fig. 4F,G). The pattern of expression observed in the circumoral nerve ring was consistent with that observed in the radial nerve cords (Fig. 4H,I). No cells expressing ArLQP were detected in the hyponeural region of the radial nerve cords and circumoral nerve ring (Fig. 4C–I).

Locomotion in *A*. *rubens* is mediated by tube feet that are located on the underside of each arm, with two rows of tube feet on either side of the radial nerve cord (Figs 4A, 5A). Cells expressing ArLQP were detected in the basal nerve ring in the disk region of tube feet (Fig. 5B,C). The digestive system of *A*. *rubens* comprises a mouth located on the underside of the central disk region, which is linked by a short oesophagus to the highly folded cardiac stomach, which is everted through the mouth during feeding. Aboral to the cardiac stomach is the pyloric stomach, which is linked to paired digestive organs (pyloric caeca) in each arm by pyloric ducts (Figs 4A, 5A). Cells expressing ArLQP were detected in the cardiac stomach and pyloric stomach, but the density of stained cells was much higher in the cardiac stomach than in the pyloric stomach (Fig. 5D,E,F). High magnification images of the cardiac stomach reveal that the stained cells are located close to the basi-epithelial nerve plexus layer and within the mucosal layer (Fig. 5E).

### ArLQ causes dose-dependent relaxation of *in vitro* preparations of tube feet from *A*. *rubens*

**Figure 6:**
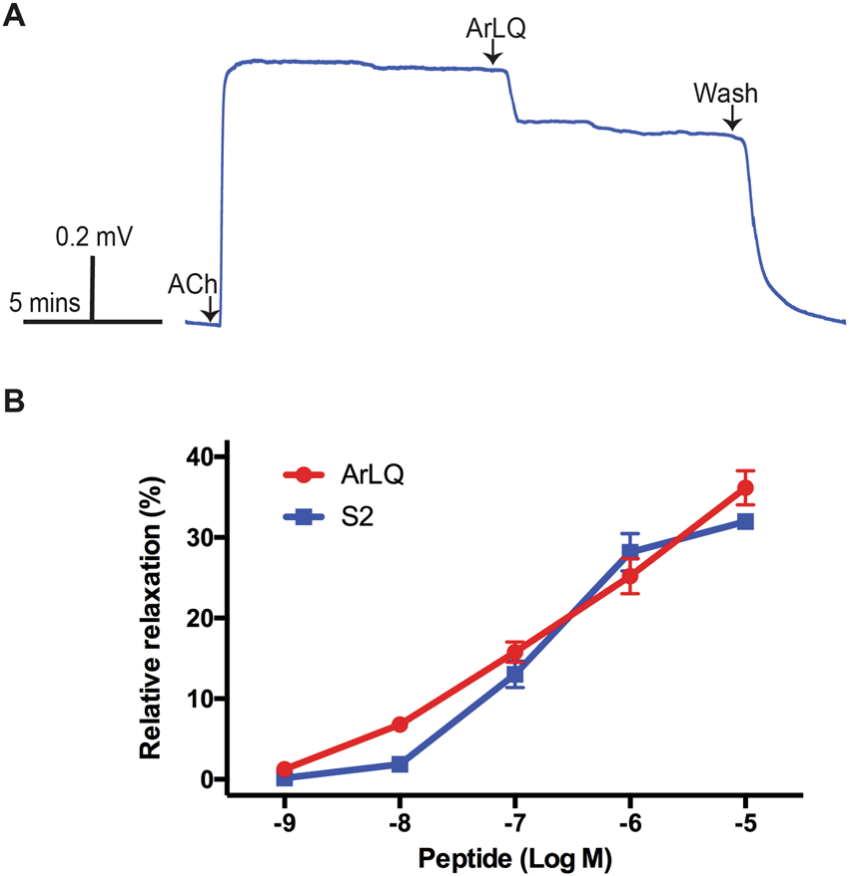
ArLQ causes relaxation of *in vitro* preparations of tube feet from *A*. *rubens*. (**A**) Representative recording of an experiment where ArLQ (1 µM) causes partial reversal of acetylcholine (ACh; 10 µM) induced contraction of an *in vitro* preparation of a tube foot from *A*. *rubens*. (**B**). Graphs showing the dose-dependent relaxing effect of ArLQ (red) on tube foot preparations in comparison with a known tube foot relaxant, the SALMFamide neuropeptide S2 (blue). Each point represents the mean ± S.E.M. from at least 6 different experiments, with the effect calculated as the percentage reversal of contraction induced by 10 μM ACh.

Informed by the pattern of expression of ArLQP in *A*. *rubens* (see above), we tested the effects of synthetic ArLQ on the contractile state of *in vitro* preparations of tube foot and cardiac stomach preparations. The rationale for this approach was that other neuropeptides that are expressed in these organs have been found to cause contraction^33^ or relaxation^27,28^ of *in vitro* preparations. Here we found that ArLQ caused relaxation of tube foot preparations that had been pre-contracted with 10 µM acetylcholine (ACh) (Fig. 6A) and the relaxing effect of ArLQ was dose-dependent when tested at concentrations ranging from 1 × 10^−9^ M to 1 × 10^−5^ M (Fig. 6B). Control tests in which 20 μl of the vehicle (water) was added to the organ bath had no effect on tube foot contractility.

Previous studies have revealed that the SALMFamide neuropeptide S2 causes relaxation of tube foot preparations from *A*. *rubens*^34^ and therefore here we compared the effects of ArLQ and S2 and found that the dose-dependence and magnitude of the effects of ArLQ were very similar to S2 (Fig. 6B). Interestingly, although expression of ArLQP was detected in the cardiac stomach of *A*. *rubens* (Fig. 5D,E), we found that when tested at concentrations ranging from 1 × 10^−9^ M to 1 × 10^−5^ ArLQ did not cause relaxation of cardiac stomach preparations, which had been pre-contracted with artificial seawater supplemented with 30 mM KCl (Supplementary Fig. 6).

## Discussion

Previous studies have reported the presence of genes encoding luqin/RYamide-type precursors and receptors in deuterostomian invertebrates^3,22,31^. Here we report the first experimental characterisation of luqin/RYamide-type neuropeptide signalling in a deuterostome – the starfish *Asterias rubens* (phylum Echinodermata).

To investigate the occurrence of luqin/RYamide-type receptors in *A*. *rubens*, we performed a comprehensive analysis of the phylogenetic distribution of luqin/RYamide-type receptors and their relationships with closely related G-protein coupled neuropeptide receptors. This was important and necessary because historically some luqin/RYamide-type neuropeptide receptors have been misnamed. For example, the first receptor for a luqin-type neuropeptide discovered in the mollusc *Lymnaea stagnalis* (GRL106) was annotated and described as a possible ortholog of vertebrate neuropeptide-Y (NPY) receptors^8^. Subsequently, the *Caenorhabditis elegans* receptor Y59H11AL.1 was annotated as a tachykinin-like receptor^35^, but a recent experimental study has demonstrated that a luqin/RYamide-type neuropeptide is the ligand for this receptor^21^. Incorporating receptor sequences from a variety of phyla, our phylogenetic analysis revealed that luqin/RYamide-type receptors form a monophyletic group of receptors that is distinct from tachykinin-type receptors and NPY-type receptors, consistent with findings from a previously reported analysis of neuropeptide receptor relationships^3^. Furthermore, we conclude that luqin/RYamide-type receptors and tachykinin-type receptors are paralogous and probably arose by gene duplication in a common ancestor of the Bilateria, with NPY-type receptors being more distantly related and occupying an outgroup position with respect to luqin/RYamide-type receptors and tachykinin-type receptors. It is noteworthy that positioned within the clade comprising luqin/RYamide-type receptors are proteins that have been annotated as NPY-type receptors, including receptors in the priapulid *Priapulus caudatus* (XP_014666446.1, XP_014678140.1) and receptors in the gastropod mollusc in which luqin was originally discovered, *Aplysia californica* (XP_012937781.1), and in the cephalopod mollusc *Octopus bimaculoides* (XP_014786450.1). Therefore, the findings of this paper provide a basis for re-annotation of these receptors as luqin/RYamide-type receptors.

Inclusion of receptor sequences from echinoderms in our phylogenetic analysis enabled identification of both luqin/RYamide-type receptors and tachykinin-type receptors in the starfish *A*. *rubens* and the sea urchin *S*. *purpuratus*. Thus, two luqin/RYamide-type receptors were identified in both *A*. *rubens* (ArLQR1 and ArLQR2) and *S*. *purpuratus*, two tachykinin-type receptors were identified in *A*. *rubens* and one tachykinin-type receptor was identified in *S*. *purpuratus*. The peptides that act as ligands for echinoderm tachykinin receptors remain to be determined, although candidate ligands have been proposed^22^. Candidate ligands for echinoderm luqin/RYamide-type receptors have also been reported, including the peptide EEKTRFPKFMRW-NH_2_ (ArLQ) in the starfish *A*. *rubens*^3,22^. Here we confirmed the structure of ArLQ by mass spectrometric analysis of an extract of radial nerve cords from *A*. *rubens*. Furthermore, we demonstrated that ArLQ acts as a ligand for both ArLQR1 and ArLQR2 when these receptors are expressed heterologously in CHO cells. Thus, the existence of a luqin/RYamide-type signalling system in a deuterostomian invertebrate has been demonstrated experimentally for the first time.

**Figure 7:**
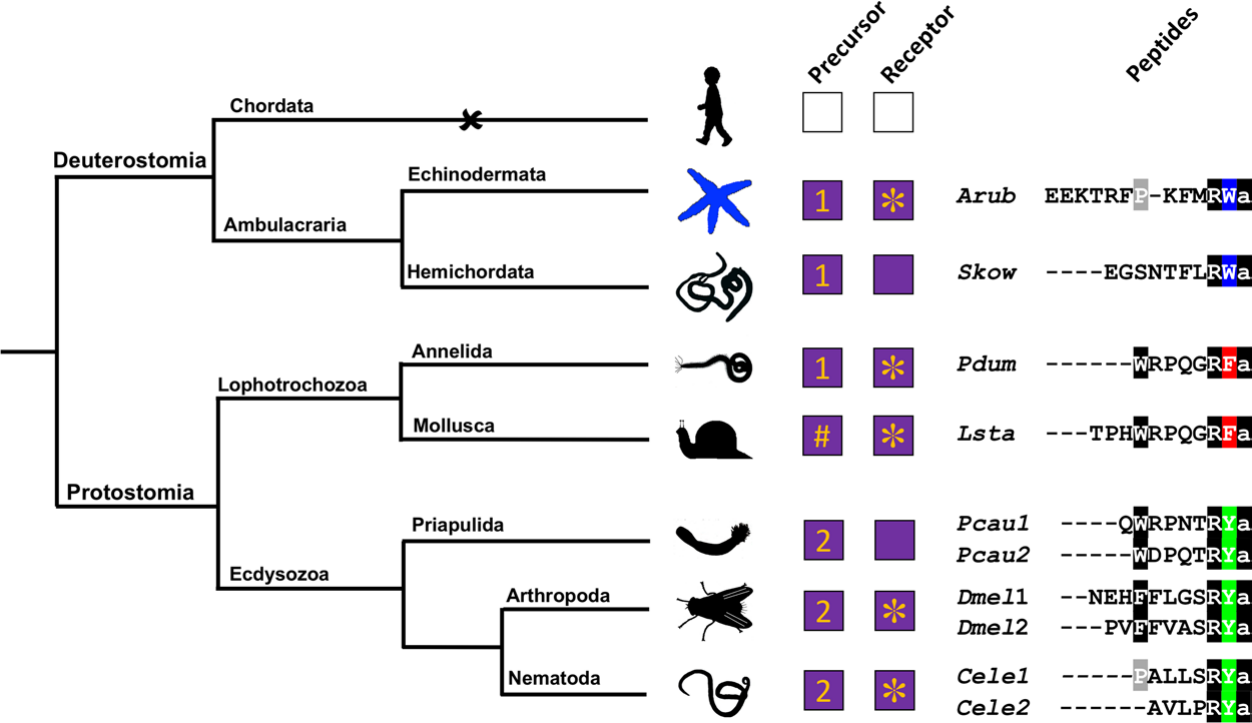
Phylogenetic diagram showing the occurrence and characteristics luqin-type neuropeptide signalling in the Bilateria. The phylogenetic tree shows relationships of selected bilaterian phyla. Phyla in which luqin-type precursors and luqin-type receptors have been identified are labelled with purple-filled boxes. The number in the precursor box indicates how many mature luqin-like neuropeptides are derived from the precursor, with a hashtag indicating mixed features. The inclusion of an asterisk in the receptor boxes indicates that the peptide ligand that activates the receptor has been determined experimentally. Note that the starfish *Asterias rubens* is the first and only deuterostome in which the neuropeptide ligand for luqin-type receptors has been identified. Note also the loss of the luqin-type signalling system in the chordate lineage, which is signified by the X and the white-filled boxes. C-terminally aligned peptides that are predicted/proven ligands for luqin-type receptors in the species listed are shown on the right side of the figure, illustrating that peptides with a C-terminal RWamide motif occur in ambulacrarians, peptides with a C-terminal RFamide motif occur in lophotrochozoans and peptides with a C-terminal RYamide motif occur in ecdysozoans. Species names are as follows: Arub (*Asterias rubens*), Skow (*Saccoglossus kowalevskii*), Pdum (*Platynereis dumerilii*), Lsta (*Lymnaea stagnalis)*, Pcau (*Priapulus caudatus*), Dmel (*Drosophila melanogaster*), Cele (*Caenorhabditis elegans*). Silhouettes of representative animals from each phylum were created by Maria Eugenia Guerra.

Characterisation of luqin/RYamide-type signalling in a deuterostome provides a basis for surveying the phylogenetic distribution and evolution of luqin/RYamide-type neuropeptide signalling, as illustrated in Fig. 7. The evolutionary origin of luqin/RYamide-type neuropeptide signalling can be traced to the common ancestor of the Bilateria, with retention in both the protostomian and deuterostomian branches of the animal kingdom. In lophotrochozoan protostomes, luqin-type neuropeptides have a C-terminal RFamide motif and cognate receptors have been characterised in molluscs and annelids^8–11,36^. In ecdysozoan protostomes, neuropeptides with a C-terminal RYamide motif have been identified as ligands for luqin/RYamide-type receptors in arthropods and nematodes^15,16,21^. Thus, we can infer that a luqin/RYamide-type neuropeptide(s) in the common ancestor of the protostomes would have had a C-terminal RFamide or RYamide motif. In the deuterostomian branch of the animal kingdom, we have identified a luqin/RYamide-type neuropeptide with a C-terminal RWamide motif as the ligand for two luqin/RYamide-type receptors in the starfish *A*. *rubens*. Closely related peptides that also have a C-terminal RWamide motif have been identified in other echinoderms, including brittle stars^31^, sea urchins^3^ and sea cucumbers^37^. Furthermore, a gene encoding a precursor protein containing a luqin/RYamide-type neuropeptide with a RWamide motif has been identified in the hemichordate *Saccoglossus kowalevskii*^3^. Thus, we can infer that a luqin/RYamide-type neuropeptide(s) in the common ancestor of the ambulacraria would have had a C-terminal RWamide motif. Interestingly, genes encoding luqin/RYamide-type receptors and precursors have not been identified in chordates (vertebrates, urochordates, cephalochordates) and therefore it can be concluded that this signalling system was lost in a common ancestor of the chordates^1,3^. In the absence of luqin/RYamide-type signalling system in chordates, we are unable to infer whether or not the RWamide motif found in ambulacrarian luqin/RYamide-type neuropeptides can be traced to the common ancestor of deuterostomes. Nor do we have sufficient information to infer the characteristics of luqin/RYamide-type neuropeptide(s) in the common ancestor of the Bilateria, with a C-terminal RWamide, RFamide or RYamide motifs all being possible.

Comparison of the structures of luqin/RYamide-type precursors in the Bilateria reveals that in some taxa the precursor contains a single luqin/RYamide-type neuropeptide, whereas in other taxa the precursor contains two or more luqin/RYamide-type neuropeptides. However, it is not possible to deduce which is the ancestral condition because in protostomes both types of precursor are found, in some cases in species belonging to the same phylum (molluscs). A highly conserved feature of luqin/RYamide-type precursors that can be traced back to the common ancestor of the Bilateria is the presence of two cysteine residues, typically separated by ten residues, located in the C-terminal region. Together with the mature luqin neuropeptide, a peptide comprising this region of the luqin precursor has been detected in extracts of neural tissue from the mollusc *A*. *californica* and named proline-rich mature peptide^38^, but its functional significance is unknown. One possibility is that this region of luqin/RYamide-type precursors is necessary for neuropeptide precursor processing during its passage through the regulated secretory pathway of neurons. Such a role has been demonstrated for cysteine-rich neurophysins, which are derived from vasopressin/oxytocin-type neuropeptide precursors and which are required for targeting of vasopressin/oxytocin-type neuropeptides to the regulated secretory pathway^39^. It is noteworthy that the two cysteine residues are highly conserved, with the exception of *Drosophila melanogaster* where the second cysteine is replaced by an arginine, which may be reflective of a functional decline of the RYamide gene in this species^18^.

Our discovery of luqin/RYamide-type signalling in *A*. *rubens* has provided a basis for the first investigation of the physiological roles of this neuropeptide system in a deuterostome. Using mRNA *in situ* hybridisation, the expression pattern of the *A*. *rubens* luqin/RYamide-type precursor (ArLQP) was examined, revealing cells in the ectoneural region of the radial nerve cords and circumoral nerve ring, in the basal nerve ring of the locomotory organs (tube foot) and in the cardiac stomach. To put this into context, this represents a relatively restricted pattern of expression by comparison with the expression patterns in *A*. *rubens* of several other neuropeptide precursors that have been analysed recently. For example, expression of gonadotropin-releasing hormone (GnRH)-type and pedal peptide/orcokinin (PP/OK)-type precursors extends to the hyponeural region of the radial nerve cords and circumoral nerve ring, the body wall and other regions of the digestive system^27,28,33^. Furthermore, it has been found that GnRH-type and PP/OK-type neuropeptides act as muscle contractants or relaxants, respectively, in *A*. *rubens*^27,28,33^. Accordingly, here we examined the effects of synthetic ArLQ on the contractility of *in vitro* preparations of organs in which ArLQP expression was detected – the tube feet and cardiac stomach. ArLQ caused dose-dependent relaxation of tube foot preparations but had no observable effect on cardiac stomach preparations. We conclude, therefore, that ArLQ may act as an inhibitory regulator of locomotory organ activity in starfish, whilst the absence of an effect of ArLQ on cardiac stomach contractility suggests that this signalling system is involved in the regulation of other aspects of stomach function in starfish.

With the first functional characterisation of luqin/RYamide-type signalling in a deuterostome, it is of interest to compare with what is known about luqin/RYamide-type neuropeptide function in protostomes. It is noteworthy that a recently reported functional analysis of luqin/RYamide-type signalling in the nematode *C*. *elegans* revealed a role in causing a reduction in locomotor activity^21^. This effect parallels our finding that ArLQ has an inhibitory effect on the contractility of locomotory organs (tube feet) in starfish. Thus, collectively these findings from a protostome (*C*. *elegans*) and a deuterostome (*A*. *rubens*) may be evidence of an evolutionary ancient role of luqin/RYamide-type signalling in regulation of locomotor activity in bilaterians. Further studies on a wider range of taxa will be required to investigate this role of luqin/RYamide-type signalling more extensively.

Like many neuropeptides, luqin/RYamide-type neuropeptides are pleiotropic, with effects on a variety of physiological processes and behaviours reported. In *C*. *elegans* luqin/RYamide-type neuropeptides inhibit feeding behaviour, an effect that is consistent with findings from arthropods, including blowflies^19^ and shrimps^20^. Furthermore, a luqin/RYamide-type neuropeptide was found to have excitatory effects on neurons and muscles involved in feeding behaviour in the mollusc *A*. *fulica*^7^. Starfish feed by everting their cardiac stomach out of their mouth and over the digestible parts of prey and therefore our finding that cells expressing ArLQP are particularly abundant in the cardiac stomach of *A*. *rubens* is consistent with the notion that luqin/RYamide-type neuropeptides may also regulate aspects of feeding behaviour in this species.

In conclusion, although the luqin precursor was first identified in the mollusc *A*. *californica* as long ago as 1986, it has attracted relatively little interest in the following three decades. With the discovery and functional characterisation of orthologs of the luqin signalling pathway in the ecdysozoa (RYamides) and in the ambulacraria (RWamides), as reported here, opportunities to gain a deeper understanding of the evolution and comparative physiology of this bilaterian neuropeptide system have emerged. The loss of luqin/RYamide/RWamide-type neuropeptides in the chordate lineage may at least in part explain why this signalling system has attracted relatively little attention, but it remains of interest to address the question of why it was lost. Furthermore, the occurrence of neuropeptide signalling systems in invertebrates that have been lost in vertebrates may have practical applications in the development of compounds that can be used to control invertebrate pests without effects on humans and other vertebrates.

## Materials and Methods

### Animals

Starfish (*A*. *rubens*) were collected at low tide from a location near Margate (Kent, UK) or were obtained from a fisherman based at Whitstable (Kent, UK). The animals were maintained in a circulating seawater aquarium at ~12 °C in the School of Biological and Chemical Sciences at Queen Mary University of London and were fed on mussels (*Mytilus edulis*) also collected near Margate.

### Cloning of a cDNA encoding the *Asterias rubens* luqin precursor and sequence alignment with other luqin/RYamide-type neuropeptide precursors

A transcript encoding the *A*. *rubens* luqin-type precursor (ArLQP) has been identified previously (GenBank: KT601719;^22^). Here a cDNA containing the complete open reading frame of ArLQP was amplified by PCR from *A*. *rubens* radial nerve cord total cDNA using specific primers (see supplementary Table 1) and Q5 proofreading polymerase (NEB; Cat. No. M0491S), cloned into pCR-Blunt II TOPO vector (Invitrogen; Cat. No. K280002) and sequenced (TubeSeq service; Eurofins Genomics). The amino acid sequence of ArLQP was aligned with luqin/RYamide-type precursors from other species (see supplementary Table 2 for a list of the sequences) using MAFFT version 7 (5 iterations, substitution matrix; BLOSUM62) and highlighted using the software BOXSHADE (www.ch.embnet.org/software/BOX_form.html) with 70% conservation as the minimum for highlighting.

### Identification of the ArLQP-derived neuropeptide ArLQ in an extract of *A*. *rubens* radial nerve cords using mass spectrometry

Radial nerve cords from two specimens of *A*. *rubens* were dissected and transferred to a micro-centrifuge tube containing 3% acetic acid (in ddH2O). The tube was incubated in a boiling water bath for 10 minutes. The nerve cords were then sonicated and homogenized to lyse cells. The extract was centrifuged, supernatant transferred to a glass vial and solvent was bubbled-off using nitrogen gas. Frozen radial nerve cord extracts were thawed and an aliquot diluted 10-fold with 0.1% aqueous formic acid, then filtered through a 0.22 μm Costar Spin-X centrifuge tube filter to remove particulates. The extract was analysed by means of nanoflow liquid chromatography with electrospray ionisation quadrupole time-of-flight tandem mass spectrometry (nanoLC-ESI-MS/MS) using a nanoAcquity UPLC® system coupled to a Synapt G2 HDMS mass spectrometer and MassLynx v4.1 SCN 908 software (Waters Corporation, Milford, MA, USA). The mobile phases used for the chromatographic separation were: 0.1% aqueous formic acid (mobile phase A) and 0.1% formic acid in acetonitrile (mobile phase B). An aliquot containing 15 μL of the extract was applied to a trapping column (Symmetry C18 180 μm × 20 mm, 5 μm particle size, 100 Å pore size, Waters Corporation) using 99.9% mobile phase A at a flow rate of 10 μL min^−1^ for 3 min, after which the fluidic flow path included the analytical capillary column (HSS T3 75 μm × 150 mm, 1.8 μm particle size, 100 Å pore size, Waters Corporation). A linear gradient of 5–40% mobile phase B over 105 min was utilized with a total run time of 120 min. Nanoflow ESI source conditions were as follows: capillary voltage 3.5 kV, sample cone voltage 25 V with a source temperature of 80 °C. The instrument was operated in resolution mode (~20,000 measured at full width half height). A data-dependent acquisition was performed that would trigger an MS/MS scan on any multiply charged peptide of intensity ≥ 450 counts/sec within the survey scan m/z range 300–1950. A maximum of 5 precursor peptides were selected for MS/MS from each survey scan and MS/MS data collected for 6 scans then combined. Each peptide precursor was then excluded from selection for MS/MS for a period of 20 sec. MS/MS data were collected over m/z range 50–1950 using m/z and charge state dependent collision energy applied to the trap region. Tandem mass spectra were extracted by ProteinLynx Global Server version 2.5.1 (Waters Corporation, Milford, MA, USA) with charge state deconvolution and deisotoping performed prior to creation of a peak list file for each sample. A peak list file generated from acetic acid extract data was used to interrogate protein database UniProtKB/TrEMBL release 2018_02 filtered for taxon identifier 7586 (phylum Echinodermata) containing 70,885 sequences and 29,368,428 residues (http://www.uniprot.org/). Search parameters used by Mascot software (Matrix Science, London, UK; version 2.5.1) were “none” for enzyme i.e. Mascot searched each protein sequence for every sub-sequence meeting the remaining search criteria, precursor mass error less than 5 ppm and fragment ion tolerance 20 mDa. A variable modification of C-terminal amidation was permitted.

### Identification of luqin/RYamide-type receptors in *A*. *rubens* and phylogenetic analysis of bilaterian luqin/RYamide-type receptors

To identify candidate receptors for ArLQ, *A*. *rubens* neural transcriptome sequence data were analysed by BLAST using the *Platynereis dumerilii* (phylum Annelida) luqin-type receptor^10^ as the query sequence. Contigs (1121303 and 1122311) containing the complete open reading frames of two luqin-type receptors were identified and the sequences of the 347 and 388 residue proteins encoded by these two transcripts were determined using the ExPASy translate tool (http://web.expasy.org/translate/) and named ArLQR1 and ArLQR2, respectively. To further investigate the relationship of ArLQR1 and ArLQR2 with luqin/RYamide*-*type receptors from other species (see supplementary Table 3 for a list of sequences), a phylogenetic analysis was performed using the maximum-likelihood method. Receptor sequences were aligned using the MUSCLE plugin in MEGA 7 (iterative, 10 iterations, UPGMB as clustering method)^40,41^ and the alignment was manually trimmed to 299 residues that span from the first to the seventh transmembrane domains. The maximum-likelihood tree was built using PhyML version 3.0 (1000 bootstrap replicates, LG substitution model)^42^.

### Pharmacological characterisation of ArLQR1 and ArLQR2

To enable pharmacological characterisation of ArLQR1 and ArLQR2, cDNAs encoding these receptors were amplified by PCR, using *A*. *rubens* radial nerve cord total cDNA, specific primers (see supplementary Table 1 for a list of the primers) and Q5 polymerase (NEB; Cat. No. M0491S). First, the receptor cDNAs were ligated into the phagemid vector pBluescript II SK + (Agilent Technologies; Cat. No. 212205) using a blunt-end ligation method. The vector was cut with the restriction enzyme *EcoRV* (NEB; Cat. No. R0195S) and the ligation was performed using T4 DNA ligase (NEB; Cat. No. M0202S). Successful ligation was confirmed by PCR, restriction enzyme digestion and sequencing (TubeSeq service; Eurofins Genomics). The receptor cDNAs were then sub-cloned into the eukaryotic expression vector pcDNA 3.1( + ) (Invitrogen; Cat. No. V790-20). To accomplish this, forward primers included the partial Kozak consensus sequence (ACC) and a sequence corresponding to the first 21 bases of the ArLQR1 or ArLQR2 ORFs, including the start codon. Reverse primers consisted of a stop codon and a sequence reverse complementary to the ORF encoding the C-terminal region of ArLQR1 or ArLQR2 (see supplementary Table 1 for a list of the primers). PCR was performed using Q5 polymerase NEB and the cDNA products were ligated into the pcDNA 3.1(+) vector that had been cut previously with the restriction enzyme *EcoRV* by performing blunt-end ligation with T4 DNA ligase NEB. The direction of the insert was determined by restriction enzyme digestion and sequencing. The ArLQR1 and ArLQR2 sequences have been deposited in the GenBank database under accession numbers MG744509 and MG744510 respectively.

To determine if ArLQ acts as a ligand for ArLQR1 and/or ArLQR2, the receptors were expressed in Chinese hamster ovary cells (CHO-K1) stably expressing the calcium-sensitive bioluminescent reporter GFP-aequorin fusion protein (G5A)^43^. CHO-K1 cells were maintained at 37 °C in T25 culture flasks (USA Scientific; Cat. No. CC7682-4325) containing 6 ml of DMEM/F12 Nutrient Mixture medium (Thermo Fisher Scientific; Cat. No. 11039047) supplemented with 10% fetal bovine serum (Thermo Fisher Scientific; Cat. No. 10082147), Antibiotic-Antimycotic 1 × (Thermo Fisher Scientific, Cat. No. 15240062) and 200 μg/ml of Geneticin G418 sulfate (Thermo Fisher Scientific, Cat. No. 10131035). Upon reaching a confluency of approximately 80%, cells were transfected with the plasmids containing ArLQR1 or ArLQR2 receptor cDNAs and a plasmid containing the promiscuous Gα-16 protein that can couple a wide range of GPCRs to the phospholipase C pathway^44^. The transfection was achieved using 5 µg of each plasmid and 8 μl of the transfection reagent Lipofectamine 3000 following the manufacturer instructions (Thermo Fisher Scientific; Cat. No. L3000008). Two days post-transfection, the culture medium was removed and the cells detached by the addition of 2 ml of 1 × PBS buffer pH 7.4 (Thermo Fisher Scientific; Cat. No. 10010023) supplemented with UltraPure EDTA 0.5 M pH 8.0 (Thermo Fisher Scientific; Cat. No. 15575020) to a final concentration of 5 mM EDTA. The cells were collected by centrifugation at 4000 rpm in an Eppendorf 5702 centrifuge (Eppendorf; Cat. No. 022626001) and the PBS-EDTA was replaced with DMEM/F12 Nutrient Mixture medium supplemented with 1 mM coelenterazine-H (Thermo Fisher Scientific; Cat. No. C6780). After an incubation period of 3 hr, cells were exposed to synthetic ArLQ peptide (EEKTRFPKFMRW-NH_2_; Peptide Protein Research Ltd., Fareham, UK) diluted in DMEM/F12 Nutrient Mixture medium in concentrations ranging from 10^−4^ M to 10^−14^ M in clear bottom 96-well plates (Sigma-Aldrich; Cat. No. CLS3603-48EA). Luminescence levels were recorded over a 35-second period using a FLUOstar Omega Plate Reader (BMG LABTECH; FLUOstar Omega Series multi-mode microplate reader). Data were integrated over the 35-second measurement period. Triplicate measurements were made for each concentration, and the average of each was used to normalise the responses. Responses were normalised to the maximum response obtained in each experiment (100% activation) and to the response obtained with the vehicle media (0% activation). Dose-response curves were fitted with a four-parameter curve and EC_50_ values were calculated from dose–response curves based on at least three measurements from three independent transfections using Prism 6 (GraphPad, La Jolla, USA).

### Localisation of ArLQP expression in *A*. *rubens* using mRNA *in situ* hybridisation

To generate probes for localisation of ArLQP expression in *A*. *rubens*, 5 μg of purified Zero Blunt® Topo vector plasmid containing the ArLQ precursor cDNA was linearised using the restriction enzymes BamH1 (NEB; Cat. No. R0136T) for the antisense probe and Xho1 (NEB; Cat. No. R0146S) for the sense probe. The linearised plasmids were cleaned by phenol:chloroform 1:1 and chloroform:isoamyl-alcohol 24:1 purification. Once purified, the DNA was precipitated by adding 1/10 volume of 3 M sodium acetate and 2.5 volumes of 100% isopropanol and incubating at −80 °C for 30 min. Following centrifugation for 10 min at 13,000 rpm in an Eppendorf 5424 R centrifuge (Eppendorf; Cat. No. 5404000138) at 4 °C, the precipitated DNA was washed with 70% ice-cold ethanol before air drying and re-suspending in RNAse-free water.

Sense and antisense RNA probes were synthesised using 1 μg of the previously linearised plasmid DNA as a template, 0.5 mM digoxigenin RNA labelling mix (Roche, Basel, Switzerland, Cat. No. 11277073910), 1 × transcription buffer (NEB, Ipswich, USA, Cat. No. M0251S), 5 mM dithiothreitol (Promega, Madison, USA, Cat. No. P1171), 1 U/μl placental ribonuclease inhibitor (NEB, Ipswich, USA, Cat. No. M0307S) and 5 U/μl of T7 RNA polymerase (NEB, Ipswich, USA, Cat. No. M0251S) for the antisense probe or 5 U/μl of SP6 RNA polymerase for the sense probe (NEB, Ipswich, USA, Cat. No. M0207S). The mixture was incubated for 2 hours at 37 °C to allow the *in vitro* transcription. The synthesised probes were purified and precipitated using the same method described for the plasmid, and stored in 25% formamide (FA)/2 × saline-sodium citrate buffer (SSC) at −20 °C. Sections of the arms and central disk of *A*. *rubens* were prepared and processed for mRNA *in situ* hybridisation using the same methods reported previously for the neuropeptide precursor ArRGPP^45^. Images of stained sections were captured using a DMRA2 light microscope (Leica) with a MicroPublisher 5.0 Real-Time Viewing (RTV) digital colour camera (QImaging) and Volocity® v.5.3.1 image analysis software (PerkinElmer). Montages of photographs were prepared using the software Adobe Photoshop CC 2015.

### Investigation of the *in vitro* pharmacological effects of ArLQ on tube foot and cardiac stomach preparations from *A*. *rubens*

Informed by findings from analysis of the expression of ArLQP in *A*. *rubens* (see results), synthetic ArLQ (custom synthesized by PPR Ltd, Fareham, UK) was tested on tube foot and cardiac stomach preparations from *A*. *rubens*. The SALMFamide neuropeptide S2 (SGPYSFNSGLTF-NH_2_) was used as control peptide for these experiments because it has been shown to as act as a relaxant of all three of these preparations^34^. Tube foot preparations were obtained by dissecting from starfish arms a small square-shaped piece of ambulacral body wall containing a single tube foot stem and its associated ampulla. Cotton ligatures were tied around the body wall and the tube foot disk, as illustrated previously^34^. Cardiac stomach preparations were dissected and prepared as previously described^46^. In both cases one ligature was attached to a fixed metal hook in a 20-ml glass organ bath containing artificial seawater at ~11 °C. The other ligature was tied to a High Grade Isotonic Transducer (ADinstruments MLT0015, Oxford, UK) connected to PowerLab data acquisition hardware (ADinstruments PowerLab 2/26, Oxford, UK). Output from PowerLab was recorded using LabChart (v8.0.7) software installed on a laptop computer (Lenovo E540, Windows 7 Professional). The preparations were left for an equilibration period of ~10 min. To enable observation of potential relaxing effects of ArLQ, tube foot preparations were pre-contracted by the addition of 10 µM acetylcholine. Once a stable baseline contracted state was reached, synthetic ArLQ or S2 (control) was added to achieve organ bath concentrations between 10^−9^ M and 10^−5^ M. In the case of the cardiac stomach, contraction was induced by using artificial seawater supplemented with 30 mM KCl, and again peptides were added once a stable baseline contracted state was reached. Cumulative dose-response curves were constructed by expressing relaxation as a percentage reversal of the contraction induced by ACh. Each peptide concentration was tested on at least four preparations from 4 different animals.

### Data availability

Data generated or analysed during this study are included in this published article or in its Supplementary Information files or are available from the corresponding author on reasonable request. The sequences of cDNAs encoding ArLQP, ArLQR1, ArLQR2, ArTKR1 and ArTKR2 have been deposited in GenBank under accession numbers KT601719, MG744509, MG744510, MG744511, MG744512, respectively.

## Electronic supplementary material

Supplementary Figures and Tables
