# Supplementary material for: Discovery and functional characterisation of a luqin-type neuropeptide signalling system in a deuterostome
Source: Sci Rep. 2018 May 8;8:7220. doi: 10.1038/s41598-018-25606-2 (PMC5940834; doi:10.1038/s41598-018-25606-2)
Supplement: Supplementary file 1 — Supplementary Figures and Tables [file 41598_2018_25606_MOESM1_ESM.pdf]

Discovery and functional characterisation of a luqin-type neuropeptide signalling system in a deuterostome

Luis Alfonso Yañez-Guerra[1], Jérôme Delroisse[1+], Antón Barreiro-Iglesias[1++], Susan E. Slade[2], James H. Scrivens[2], and Maurice R. Elphick[1]*

* Correspondence to: Prof. Maurice R. Elphick at m.r.elphick@qmul.ac.uk

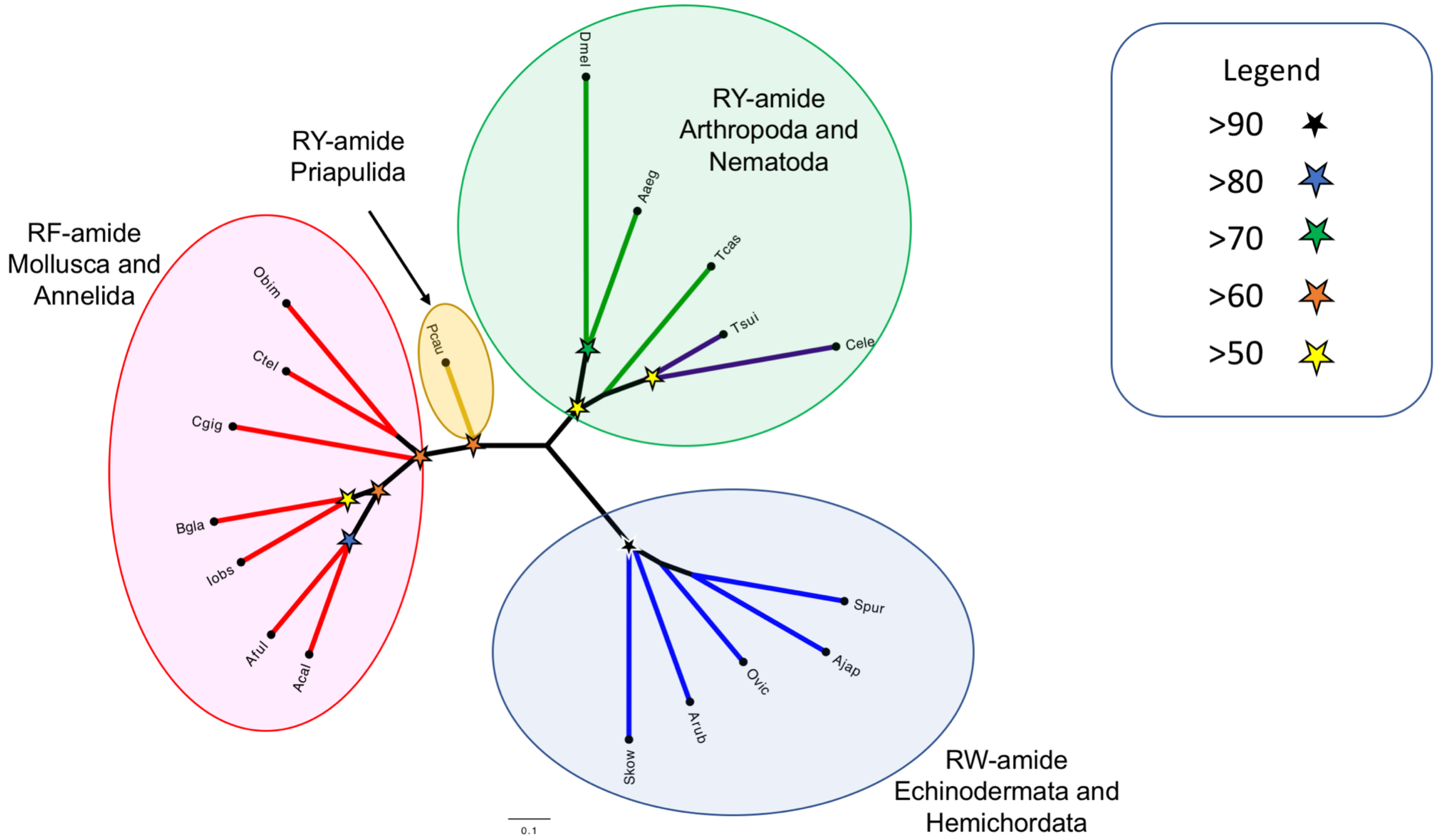

Supplementary figure 1. Neighbour-joining tree[1] showing relationships of Luqin, RYamide, and RWamide precursors. The percentage of replicate trees in which the associated taxa clustered together in the bootstrap test (5000 replicates) are shown next to the branches and are represented with coloured stars, as explained in the key. The analysis was conducted in MEGA 7[2]. Species names are as follows; Aaeg (*Aedes aegypti*), Acal (*Aplysia californica*), Aful (*Achatina fulica*), Ajap (*Apostichopus japonicus*), Arub (*Asterias rubens*), Bgla (*Biomphalaria glabrata*), Cele (*Caenorhabditis elegans*), Cgig (*Crassostrea gigas*), Ctel (*Capitella teleta*), Dmel (*Drosophila melanogaster*), Iobs (*Ilyanasa obsoleta*), Obim (*Octopus bimaculoides*), Ovic (*Ophionotus victoriae*), Pcau (*Priapulus caudatus*), Skow (*Saccoglossus kowalevskii*), Spur (*Strongylocentrotus purpuratus*), Tcas (*Tribolium castaneum*), Tsui (*Trichuris suis*). The accession numbers of the sequences included in this tree are shown in supplementary table 2.

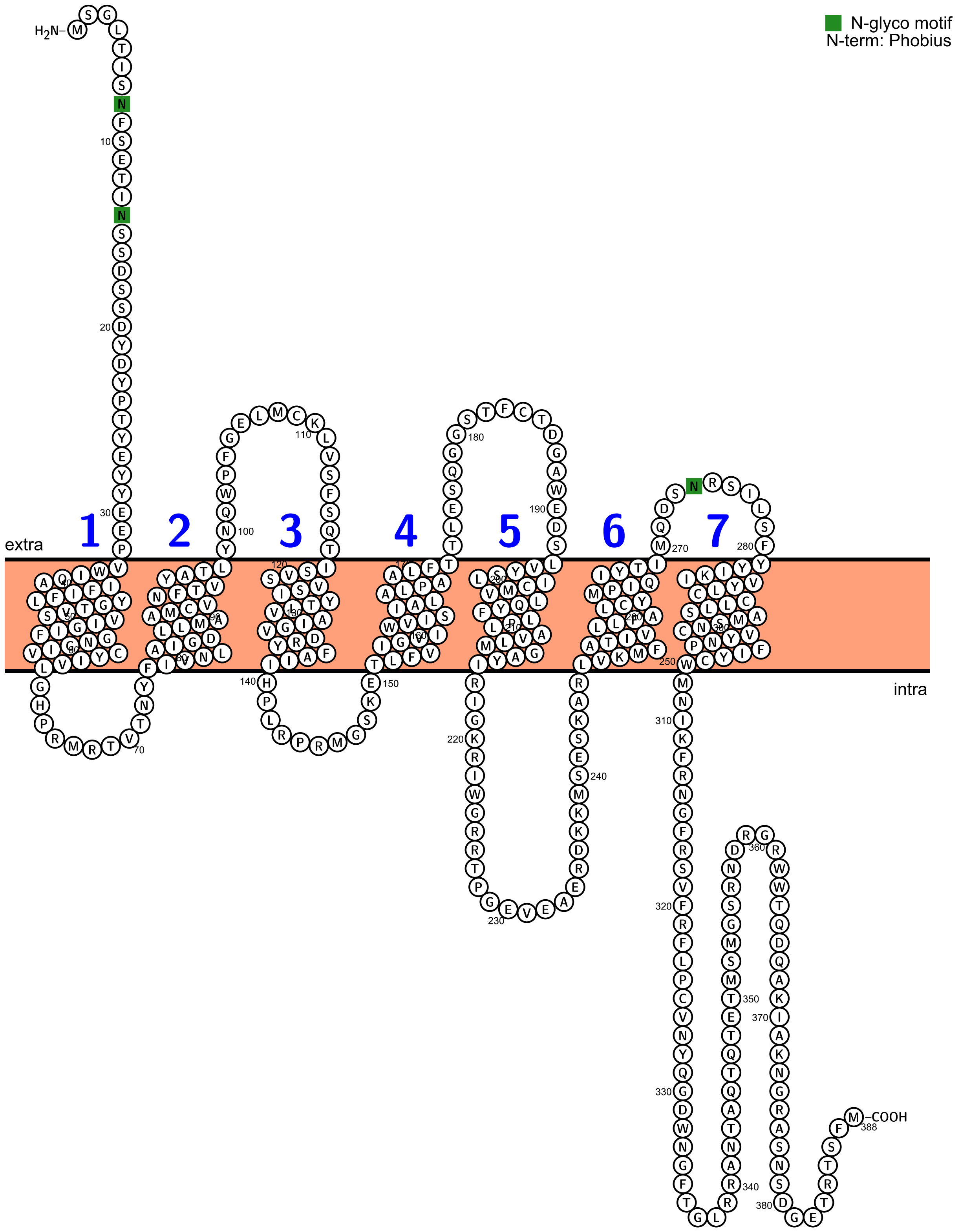

Supplementary figure 2. Prediction of the transmembrane domains of ArLQR1. The seven transmembrane domains are numbered successively in blue and N-glycosylation sites are shown with green boxes. *In silico* analysis of the amino acid sequence of this receptor was made using Protter[3].

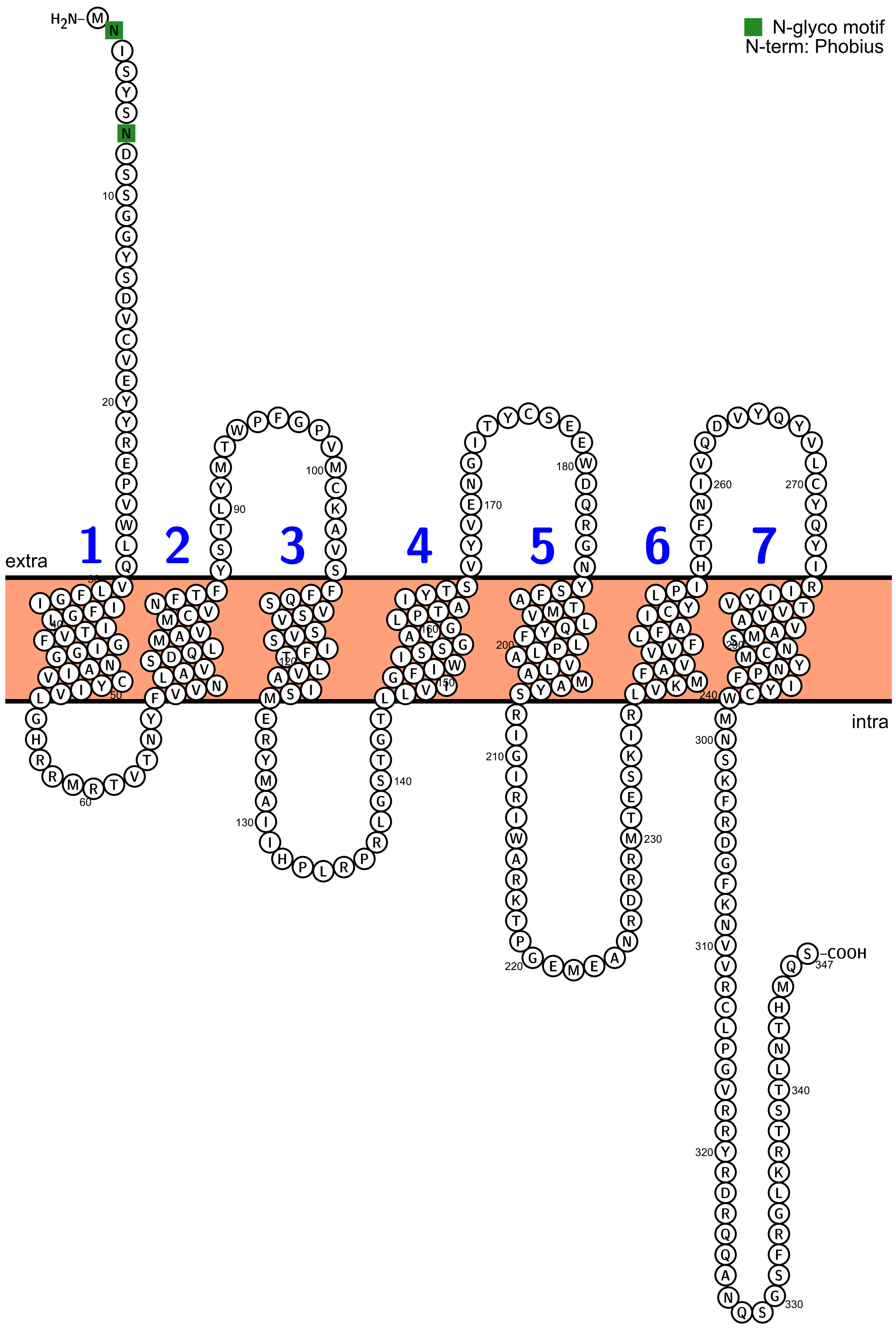

Supplementary figure 3. Prediction of the transmembrane domains of ArLQR2. The seven transmembrane domains are numbered successively in blue and N-glycosylation sites are shown with green boxes. *In silico* analysis of the amino acid sequence of this receptor was made using Protter[3].

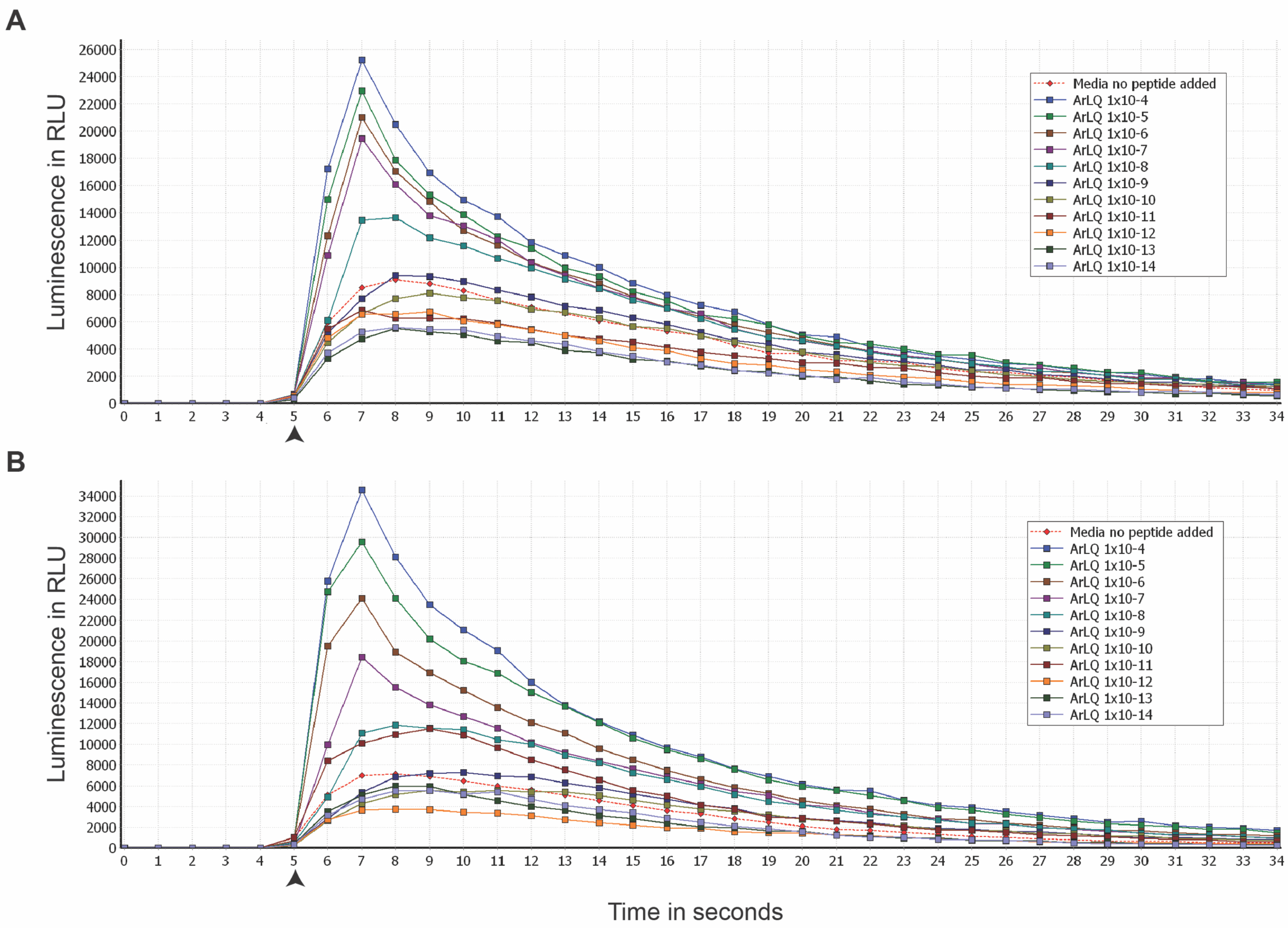

Supplementary figure 4. Graphs showing the dose-dependence and kinetics of ArLQ activation of ArLQR1 (**A**) and ArLQR2 (**B**) in a representative experiment. Luminescence was recorded over a period of 35 seconds, with injection of receptor-expressing CHO cells occurring in the 5th second of the experiment, as labelled with an arrow. The concentrations of the ArLQ peptide tested range from $1x10^{-4}$ to $1x10^{-14}$, as shown in the key.

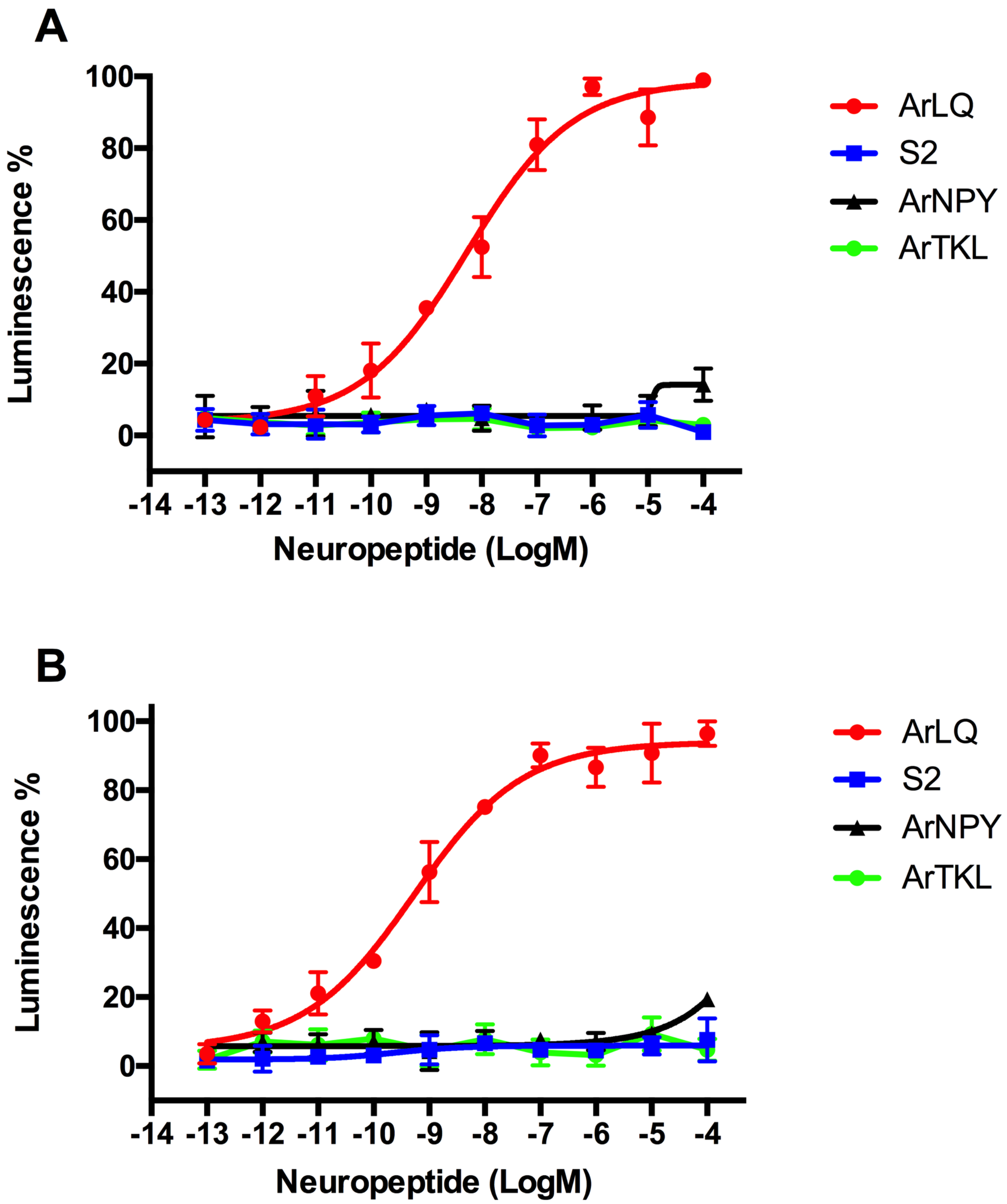

Supplementary figure 5. Graphs showing the selectivity of ArLQR1 (A) and ArLQR2 (B) as receptors for ArLQ. Thus, ArLQ causes dose-dependent activation of both receptors, whereas other starfish neuropeptides tested do not activate the receptors. Key: S2, SALMFamide-2; ArNPY, *A. rubens* neuropeptide-Y-type peptide; ArTKL, *A. rubens* tachykinin-like peptide.

Best model: LG +G+I+F

```
Substitution model                          : LG
Equilibrium frequencies                     : Empirical
Proportion of invariable sites              : estimated (0.020)
Number of substitution rate categories      : 4
Gamma shape parameter                       : estimated (1.087)
```

Model	Decoration	K	Llk	AIC	BIC
LG	+G+I+F	110	-20552,59191	41325,18382	41740,04234
LG	+G+F	109	-20557,59324	41333,18648	41744,27356
LG	+G+I	91	-20607,61452	41397,22904	41740,43018
LG	+G	90	-20618,81173	41417,62346	41757,05316
WAG	+G+I+F	110	-20620,02046	41460,04092	41874,89944
VT	+G+I+F	110	-20621,45882	41462,91764	41877,77616
JTT	+G+I+F	110	-20639,37772	41498,75544	41913,61396
CpREV	+G+I+F	110	-20642,41118	41504,82236	41919,68088
MtZoa	+G+I+F	110	-20691,79631	41603,59262	42018,45114
Blosum62	+G+I+F	110	-20716,06827	41652,13654	42066,99506
CpREV	+G+I	91	-20757,19726	41696,39452	42039,59566
RtREV	+G+I+F	110	-20742,06967	41704,13934	42118,99786
Dayhoff	+G+I+F	110	-20762,64613	41745,29226	42160,15078
DCMut	+G+I+F	110	-20763,52532	41747,05064	42161,90916
MtZoa	+G+I	91	-20812,20920	41806,41840	42149,61954
MtREV	+G+I+F	110	-20891,45977	42002,91954	42417,77806
MtArt	+G+I+F	110	-20948,01828	42116,03656	42530,89508
Flu	+G+I+F	110	-21004,75070	42229,50140	42644,35992
HIVb	+G+I+F	110	-21005,52572	42231,05144	42645,90996
MtArt	+G+I	91	-21200,92921	42583,85842	42927,05956
AB	+G+I+F	110	-21196,30234	42612,60468	43027,46320
MtMam	+G+I+F	110	-21335,22491	42890,44982	43305,30834
HIVw	+G+I+F	110	-21492,59655	43205,19310	43620,05162

Supplementary figure 6. Determination of the best amino acid substitution model for maximum-likelihood based phylogenetic analysis of the receptors analysed in this study. Amino acid substitution models are shown in descending order, with the best model corresponding to the LG substitution model. This analysis was conducted using PhyML [45,6]

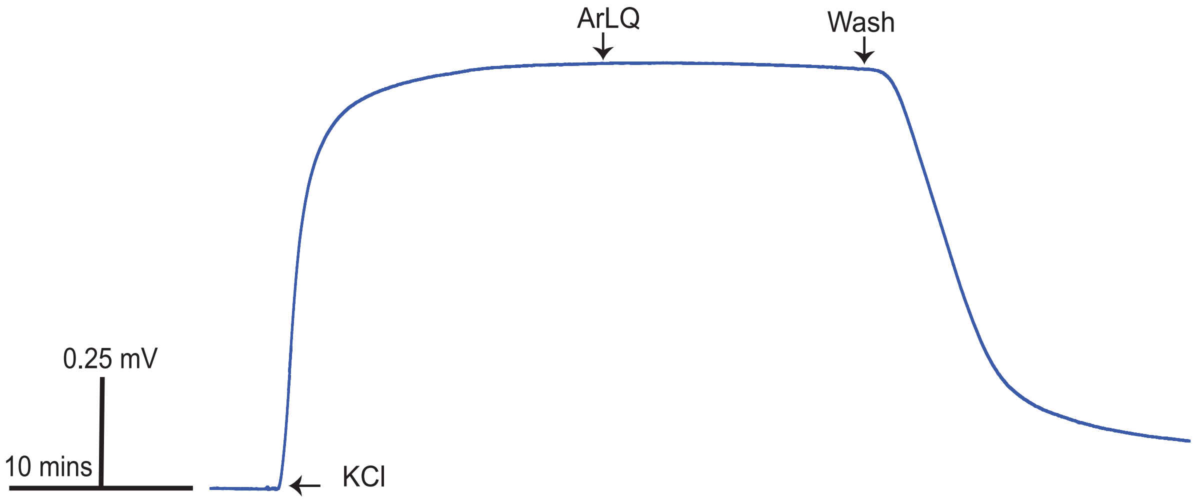

Supplementary figure 7. ArLQ does not cause relaxation of *in vitro* preparations of cardiac stomach from *A. rubens*. Representative recording of an experiment where ArLQ (1 µM) was added after the induced contraction (KCl) of an *in vitro* preparation of a cardiac stomach from *A. rubens*.

**Primers used for cloning of ArLuqin precursor cDNA**			
	Sequence	*Length*	*Calculated melting temperature ºC*
Forward primer 5' to 3'	TAGTCGGTGTGAAGGCTCTG	20	56.4
Reverse primer 5' to 3'	AGATGTCTCGTCGTTTCGGT	20	56
**Primers used for cloning of ArLuqin receptor 1 (ArLQR1) cDNA in pBlueScript II SK (+)**			
	Sequence	*Length (pb)*	*Calculated melting temperature ºC*
Forward primer 5' to 3'	ATGTCGGGATTAACAATATC	20	57
Reverse primer 5' to 3'	TCACATGAAAGATGTTCTTGT	21	57.3
**Primers used for cloning of ArLuqin receptor 1 (ArLQR1) cDNA in pcDNA3.1 +**			
	Sequence	*Length (pb)*	*Calculated melting temperature ºC*
Forward primer 5' to 3'	**ACCATG**TCGGGATTAACAA	19	60.1
Reverse primer 5' to 3'	TCACATGAAAGATGTTCTTGT	21	57.3
**Primers used for cloning of ArLuqin receptor 2 (ArLQR2) cDNA in pBlueScript II SK (+)**			
	Sequence	*Length*	*Calculated melting temperature ºC*
Forward primer 5' to 3'	ATGAATATATCGTACAGCAA	20	52
Reverse primer 5' to 3'	TCAGGATTGCATGTGTGTATT	21	60
**Primers used for cloning of ArLuqin receptor 2 (ArLQR2) cDNA in pcDNA3.1 +**			
	Sequence	*Length*	*Calculated melting temperature ºC*
Forward primer 5' to 3'	**ACCATG**AATATATCGTACAGC	21	56.5
Reverse primer 5' to 3'	TCAGGATTGCATGTGTGTATT	21	60
	**ACCATG*** Partial kozak sequence used for expression in mammalian cells		

Supplementary Table 1. Primers used for cloning of cDNAs encoding ArLQP, ArLQR1 and ArLQR2.

Abbreviation	Species name	Accesion number	Protein	Taxa	Phyla
Arub	*Asterias rubens*	ALJ99961.1	Luqin-type	Deuterostome	Ambulacraria (echinoderm)
Ovic	*Ophionotus victoriae*	MF155242.1	Luqin-type	Deuterostome	Ambulacraria (echinoderm)
Ajap	*Apostichopus japonicus*	ISOTIG 13831	Luqin-type	Deuterostome	Ambulacraria (echinoderm)
Spur	*Strongylocentrotus purpuratus*	XP_003723362.1	Luqin-type	Deuterostome	Ambulacraria (echinoderm)
Skow	*Saccoglossus kowalevskii*	XR_438635.1	Luqin-type	Deuterostome	Ambulacraria (hemichordate)
Ctel	*Capitella teleta*	ELU01624.1	Luqin-type	Protostome	Lophotrochozoa (annelid)
Pdur	*Platynereis durmerilii*	AHB62380.1	Luqin-type	Protostome	Lophotrochozoa (annelid)
Obim	*Octopus bimaculoides*	XP_014774083.1	Luqin-type	Protostome	Lophotrochozoa (mollusc)
Cgig	*Crassostea gigas*	XP_011447715.1	Luqin-type	Protostome	Lophotrochozoa (mollusc)
Acal	*Aplysia californica*	NP_001191480.1	Luqin	Protostome	Lophotrochozoa (mollusc)
Aful	*Achatina fulica*	BAA76406.1	Luqin-type	Protostome	Lophotrochozoa (mollusc)
Iobs	*Ilyanasa obsoleta*	FK719020.1	Luqin-type	Protostome	Lophotrochozoa (mollusc)
Bgla	*Biomphalaria glabrata*	XP_013066378.1	Luqin-type	Protostome	Lophotrochozoa (mollusc)
Pcau	*Priapulus caudatus*	XP_014674262.1	RYamide	Protostome	Ecdysozoa (priapulid)
Tcas	*Tribolium castaneum*	NP_001280530.1	RYamide	Protostome	Ecdysozoa (arthropod)
Dmel	*Drosophila melanogaster*	NP_001104382.3	RYamide	Protostome	Ecdysozoa (arthropod)
Aaeg	*Aedes aegypti*	XP_001655654.1	RYamide	Protostome	Ecdysozoa (arthropod)
Tsui	*Trichuris suis*	KFD52143.1	RYamide	Protostome	Ecdysozoa (nematode)
Cele	*Caenorhabditis elegans*	NP_001255160.1	RYamide	Protostome	Ecdysozoa (nematode)

Supplementary Table 2. Accession numbers of the neuropeptide precursor sequences used for the alignment in Figure 1 and Supplementary Figure 1. Ambulacrarian sequences are displayed with blue colour, Lophotrochozoan sequences with red colour and Ecdysozoan sequences with green colour.

Abbreviation (Species name)	Receptor type	Accession numbers
Arub (Asterias rubens)	Luqin	MG744509, MG744510
Spur (Strongylocentrotus purpuratus)	Luqin	XP_783326.1, XP_783390.1
Skow (Saccoglossus kowalevskii)	Luqin	XM_002731957.1, XM_002731958.1, XM_006813011.1, XM_002731956.1
Acal (Aplysia californica)	Luqin	XP_012937781.1
Lgig (Lottia gigantea)	Luqin	XP_009064514.1, XP_009064591.1
Lsta (Lymnea stagnalis)	Luqin	AAB92258.1
Obim (Octopus bimaculoides)	Luqin	XP_014786450.1
Ctel (Capitella teleta)	Luqin	ELT96089.1
Pdum (Platynereis dumerilii)	Luqin	KP420214.1
Pcau (Priapulus caudatus)	Luqin	XP_014666446.1, XP_014678140.1
Apis (Acyrthosiphon pisum)	RYamide	XP_008178727.1, XP_003241610.1
Tcas (Tribolium castaneum)	RYamide	HQ709383.1
Aae (Aedes aegypti)	RYamide	AGX85003.1
Dmel (Drosophila melanogaster)	RYamide	P25931.2
Cele (Caenorhabditis elegans)	Luqin	NP_001023541.1
Tsui (Trichuris suis)	Luqin	KFD65303.1
Hsap (Homo sapiens)	Tachykinin	AAB20303.1, NP_001049.1, NP_001050.1
Cint (Ciona intestinalis)	Tachykinin	XM_009863501.2
Arub (Asterias rubens)	Tachykinin	MG744511, MG744512
Spur (Strongylocentrotus purpuratus)	Tachykinin	XP_011662258.1
Ovul (Octopus vulgaris)	Tachykinin	BAD93354.1
Acal (Aplysia californica)	Tachykinin	XP_012936180.1
Lgig (Lottia gigantea)	Tachykinin	XP_009062052.1
Ctel (Capitella teleta)	Tachykinin	ELT98449.1
Uuni (Urechis unitinctus)	Tachykinin	BAB87199.1
Dmel (Drosophila melanogaster)	Tachykinin	FBtr0085507
Tcas (Tribolium castaneum)	Tachykinin	XP_008194527.2
Hsap (Homo sapiens)	Neuropeptide Y/F	NP_001265724.1, NP_000900.1, NP_001304020.1
Spur (Strongylocentrotus purpuratus)	Neuropeptide Y/F	XP_003725178.1
Lymnaea stagnalis	Neuropeptide Y/F	CAA57620.1
Pdum (Platynereis dumerilii)	Neuropeptide Y/F	AKQ63001.1
Dmel (Drosophila melanogaster)	Neuropeptide Y/F	AAF51909.3
Hsap (Homo sapiens)	TRH	NP_003292.1
Pdum (Platynereis dumerilii)	TRH	AKQ63029.1
Pcau (Priapulus caudatus)	TRH	XP_014663378.1
Dpul (Daphnia pulex)	TRH	ADZ15312.1

Supplementary Table 3. Accession numbers of the receptor sequences used for the phylogenetic tree in Figure 2.

Luqin Receptor<br>*Platynereis dumerilii*	Bauknecht, P. & Jékely, G. Large-Scale Combinatorial Deorphanization of Platynereis Neuropeptide GPCRs. *Cell Rep* **12,** 684–693 (2015).
Luqin Receptor<br>*Lymnaea stagnalis*	Tensen, C. P. *et al.* The lymnaea cardioexcitatory peptide (LyCEP) receptor: a G-protein-coupled receptor for a novel member of the RFamide neuropeptide family. *J Neurosci* **18,** 9812–9821 (1998).
RYamide Receptor<br>*Drosophila melanogaster*	Ida, T. *et al.* Identification of the novel bioactive peptides dRYamide-1 and dRYamide-2, ligands for a neuropeptide Y-like receptor in Drosophila. *Biochem Biophys Res Commun* **410,** 872–877 (2011).
RYamide Receptor<br>*Tribolium castaneum*	Collin, C. *et al.* Identification of the Drosophila and Tribolium receptors for the recently discovered insect RYamide neuropeptides. *Biochem Biophys Res Commun* **412,** 578–583 (2011).
Luqin Receptor<br>*Caenorhabditis elegans*	Ohno, H. *et al.* Luqin-like RYamide peptides regulate food-evoked responses in C. elegans. *elife* **6,** (2017).
Tachykinin Receptors<br>*Homo sapiens* NK1-3	Takeda, Y., Chou, K. B., Takeda, J., Sachais, B. S. & Krause, J. E. Molecular cloning, structural characterization and functional expression of the human substance P receptor. *Biochem Biophys Res Commun* **179,** 1232–1240 (1991).<br>Laburthe, M., Couvineau, A., Amiranoff, B. & Voisin, T. Receptors for gut regulatory peptides. *Baillieres Clin Endocrinol Metab* **8,** 77–110 (1994)<br>Kurtz, M. M. *et al.* Identification, localization and receptor characterization of novel mammalian substance P-like peptides. *Gene* **296,** 205–212 (2002)<br>Lecci, A., Capriati, A., Altamura, M. & Maggi, C. A. Tachykinins and tachykinin receptors in the gut, with special reference to NK2 receptors in human. *Auton Neurosci* **126-127,** 232–249 (2006).
Tachykinin Receptor<br>*Ciona intestinalis*	Satake, H. *et al.* Tachykinin and tachykinin receptor of an ascidian, Ciona intestinalis: evolutionary origin of the vertebrate tachykinin family. *J Biol Chem* **279,** 53798–53805 (2004).
Tachykinin Receptor<br>*Octopus vulgaris*	Kanda, A., Takuwa-Kuroda, K., Aoyama, M. & Satake, H. A novel tachykinin-related peptide receptor of Octopus vulgaris-evolutionary aspects of invertebrate tachykinin and tachykinin-related peptide. *FEBS J* **274,** 2229–2239 (2007).
Tachykinin Receptor<br>*Urechis unicinctus*	Kawada, T. *et al.* A novel tachykinin-related peptide receptor. Sequence, genomic organization, and functional analysis. *Eur J Biochem* **269,** 4238–4246 (2002).
Tachykinin Receptor<br>*Drosophila melanogaster*	Li, X. J., Wolfgang, W., Wu, Y. N., North, R. A. & Forte, M. Cloning, heterologous expression and developmental regulation of a Drosophila receptor for tachykinin-like peptides. *EMBO J* **10,** 3221–3229 (1991).
Neuropeptide Y<br>Receptors 1, 4, 5<br>*Homo sapiens*	Tatemoto, K., Carlquist, M., Mutt, V. 1982. Neuropeptide V-A novel brain peptide with structural similarities to peptide YY and pancreatic polypeptide. Nature 296:659-60<br>Wahlestedt, C. and Reis, D.J. (1993) Annu. Rev. Pharmacol. Toxicol. 32, 309-352<br>Grundemar, L., Sheikh, S.P. and Wahlestedt, C. (1993) in: The Biology of Neuropeptide Y and Related Peptides, Humana Press Inc. (Totawa, New Jersey), pp. 197-239.<br>Bard, J. A., Walker, M. W., Branchek, T. A. & Weinshank, R. L. Cloning and functional expression of a human Y4 subtype receptor for pancreatic polypeptide, neuropeptide Y, and peptide YY. *J Biol Chem* **270,** 26762–26765 (1995).
Neuropeptide Y receptor<br>*Lymnaea stagnalis*	Tensen, C. P. *et al.* Molecular cloning and characterization of an invertebrate homologue of a neuropeptide Y receptor. *Eur J Neurosci* **10,** 3409–3416 (1998).
Neuropeptide Y receptor<br>*Platynereis dumerilii*	Bauknecht, P. & Jékely, G. Large-Scale Combinatorial Deorphanization of Platynereis Neuropeptide GPCRs. *Cell Rep* **12,** 684–693 (2015).
Neuropeptide F receptor<br>*Drosophila melanogaster*	Garczynski SF, Brown MR, Shen P, Murray TF, Crim JW (2002) Characterization of a functional neuropeptide F receptor from Drosophila melanogaster. Peptides 23: 773-780. doi:10.1016/ S0196-9781(01)00647-7. PubMed: 11897397.

Supplementary Table 4. List of references that report deorphanisation of the receptors that are highlighted in blue in Figure 2.

Bibliography

1. Saitou, N. & Nei, M. The neighbor-joining method: a new method for reconstructing phylogenetic trees. *Mol Biol Evol* **4,** 406–425 (1987).

2. Kumar, S., Stecher, G. & Tamura, K. MEGA7: molecular evolutionary genetics analysis version 7.0 for bigger datasets. *Mol Biol Evol* **33,** 1870–1874 (2016).

3. Omasits, U., Ahrens, C. H., Müller, S. & Wollscheid, B. Protter: interactive protein feature visualization and integration with experimental proteomic data. *Bioinformatics* **30,** 884–886 (2014).

4. Lefort, V., Longueville, J.-E. & Gascuel, O. SMS: smart model selection in phyml. *Mol Biol Evol* **34,** 2422–2424 (2017).

5. Guindon, S., Delsuc, F., Dufayard, J.-F. & Gascuel, O. Estimating maximum likelihood phylogenies with PhyML. *Methods Mol Biol* **537,** 113–137 (2009).

6. Guindon, S. *et al.* New algorithms and methods to estimate maximum-likelihood phylogenies: assessing the performance of PhyML 3.0. *Syst Biol* **59,** 307–321 (2010).
